# A Phase Ia Study to Assess the Safety and Immunogenicity of New Malaria Vaccine Candidates ChAd63 CS Administered Alone and with MVA CS

**DOI:** 10.1371/journal.pone.0115161

**Published:** 2014-12-18

**Authors:** Eoghan de Barra, Susanne H. Hodgson, Katie J. Ewer, Carly M. Bliss, Kerrie Hennigan, Ann Collins, Eleanor Berrie, Alison M. Lawrie, Sarah C. Gilbert, Alfredo Nicosia, Samuel J. McConkey, Adrian V. S. Hill

**Affiliations:** 1 Royal College of Surgeons in Ireland, Dublin, Ireland; 2 Centre for Clinical Vaccinology and Tropical Medicine, University of Oxford, Churchill Hospital, Oxford, United Kingdom; 3 The Jenner Institute, University of Oxford, Old Road Campus Research Building, Oxford, United Kingdom; 4 Clinical Biomanufacturing Facility, University of Oxford, Churchill Hospital, Oxford, United Kingdom; 5 Okairòs AG, Rome, Italy; 6 CEINGE, Naples, Italy; Sanaria. Inc, United States of America

## Abstract

**Background:**

Plasmodium falciparum (P. falciparum) malaria remains a significant cause of mortality and morbidity throughout the world. Development of an effective vaccine would be a key intervention to reduce the considerable social and economic impact of malaria.

**Methodology:**

We conducted a Phase Ia, non-randomized, clinical trial in 24 healthy, malaria-naïve adults of the chimpanzee adenovirus 63 (ChAd63) and modified vaccinia virus Ankara (MVA) replication-deficient viral vectored vaccines encoding the circumsporozoite protein (CS) of *P. falciparum*.

**Results:**

ChAd63-MVA CS administered in a heterologous prime-boost regime was shown to be safe and immunogenic, inducing high-level T cell responses to CS. With a priming ChAd63 CS dose of 5×10^9^ vp responses peaked at a mean of 1947 SFC/million PBMC (median 1524) measured by ELIspot 7 days after the MVA boost and showed a mixed CD4+/CD8+ phenotype. With a higher priming dose of ChAd63 CS dose 5×10^10^ vp T cell responses did not increase (mean 1659 SFC/million PBMC, median 1049). Serum IgG responses to CS were modest and peaked at day 14 post ChAd63 CS (median antibody concentration for all groups at day 14 of 1.3 µg/ml (range 0–11.9), but persisted throughout late follow-up (day 140 median antibody concentration groups 1B & 2B 0.9 µg/ml (range 0–4.7).

**Conclusions:**

ChAd63-MVA is a safe and highly immunogenic delivery platform for the CS antigen in humans which warrants efficacy testing.

**Trial Registration:**

ClinicalTrials.gov NCT01450280

## Introduction

Plasmodium falciparum (*P. falciparum*) malaria remains a cause of significant mortality and morbidity throughout the world [1]. Though one vaccine, RTS,S, has demonstrated promising results in Phase III trials, there remains a need to develop an alternative, more effective vaccine. For more than 40 years it has been known that it is possible to achieve high-level, sustained, protective immunity against the pre-erythrocytic stages of *P. falciparum* infection through immunization with the bites of >1000 infected, irradiated mosquitoes [2]–[5].

For many years the evidence suggested that antibodies against the major sporozoite surface antigen, the circumsporozoite protein (CS), were responsible for protection and this formed the basis of the design of the RTS,S vaccine [6]. However, based on data from murine adoptive transfer experiments and human trials it now seems that CD8^+^ T cells specific for parasite-derived peptide/class I MHC molecule complexes on the surface of infected hepatocytes are the primary immune effectors [7]–[14]. Thus the goal in malaria vaccine development is a vaccine that induces both humoral and cell-mediated immune responses resulting in memory T and B cells that are specific for epitopes derived from parasite proteins. Initially, it was thought that cytolysis of the infected hepatocyte by parasite-specific CD8^+^ T cells was the primary effector mechanism, but recent data suggest that the elimination of the infected hepatocytes is mediated by interferon-gamma (IFN-γ) released by CD8^+^ T cells [15]. Researchers at the University of Oxford have been working for over 10 years to develop a pre-erythrocytic *P. falciparum* malaria vaccine using the sporozoite and liver stage antigen ME-TRAP. This antigen contains a fusion protein of multiple epitopes (ME: a string of 20 epitopes, mainly CD8+ T cell epitopes from pre-erythrocytic antigens) and the *P. falciparum* pre-erythrocytic antigen thrombospondin-related adhesion protein (TRAP) [16]. Multiple vectors for this antigen have been clinically tested including DNA, fowl pox (FP) and modified vaccinia virus Ankara (MVA), however T cell immunogenicity and clinical efficacy has been limited [17]–[19]. More recently, heterologous prime boost with Chimpanzee adenovirus 63 (ChAd63) and MVA, both expressing ME-TRAP, has been shown to be the most immunogenic regimen to date, inducing more than 2400 IFNγ producing T cells post boost [20]–[22]. This heterologous prime-boost regime with the viral vectors ChAd63 and MVA has been shown to induce the highest T cell responses in humans of any vaccine platform, as well as strong antibody responses [23]–[25]. Simian adenoviruses are not known to cause pathology or illness in humans and the prevalence of antibodies to chimpanzee origin adenoviruses is less than 5% in humans residing in the USA [26]. In Equatorial Africa prevalence is higher. A recent study in Kenya showed 4% of children to have high neutralising antibodies to ChAd63 [27]. The presence of pre-existing antibodies to adenoviral vectors has been an issue with human adenoviral vectors. However, data from the Phase IIb efficacy study of ChAd63-MVA ME-TRAP showed no correlation between neutralising antibodies to ChAd63 in volunteers prior to vaccination with their subsequent T cell count post MVA boost, suggesting that even if neutralising antibodies exist they may not limit immunogenicity [28]. The ChAd63 vector is replication deficient as the essential E1 gene region has been deleted and the virus only propagates in cells expressing E1 functions. This means the virus will not replicate in human cells within the body. Pre-clinical bioavailability studies have demonstrated no persistence of the ChAd63 vector 24 hours post intramuscular administration. ChAd63 expressing various antigens has been administered to over 400 individuals including children and has demonstrated an excellent safety profile. Multiple studies have shown 5×10^10^ vp ChAd63 to be the optimal dose [20], [21], [23], [28], [29].

With this platform the immune system is primed with a simian adenovirus expressing an antigen and then boosted 8 weeks later with Modified Vaccinia Ankara (MVA) expressing the same antigen. Several antigens have been trialled using this platform [20], [22], [29] including ME-TRAP, which has shown sterile protection in 21% of malaria-naïve volunteers in controlled human malaria infection (CHMI) [28]. In this study we combined this platform with the circumsporozoite protein (CSP).

## Methods

### Objective

**Figure 1:**
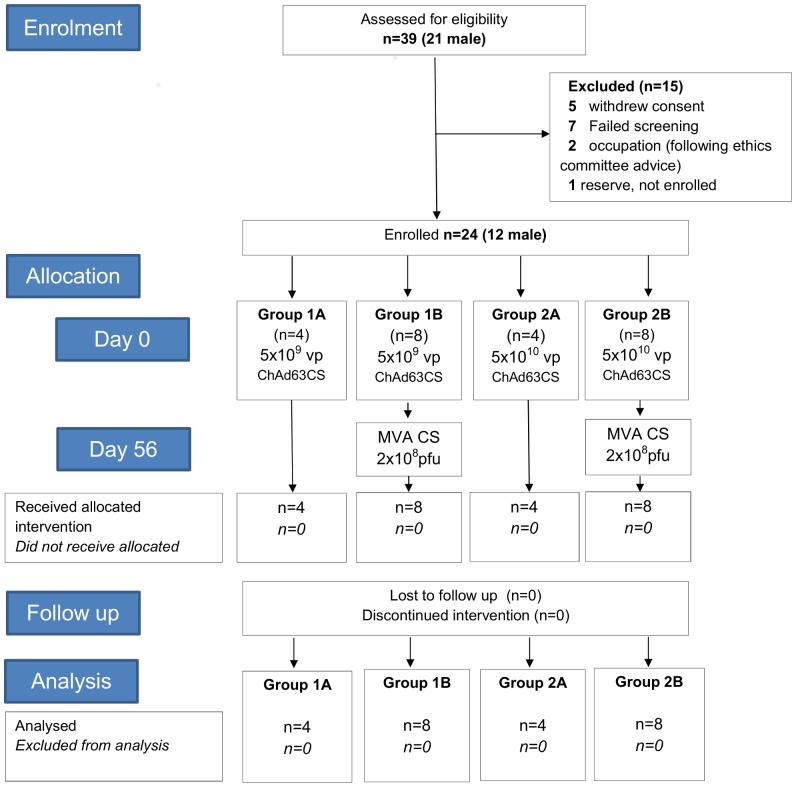
CONSORT diagram of study progress. 39 volunteers were screened. Reasons for not meeting the inclusion criteria in 7 excluded volunteers were: psychiatric morbidity (2), history of malignancy (2), one each of: history of headaches, Carbohydrate Deficient Transferrin (CDT) >3% and neutropenia.

The objective of the study was to assess the reactogenicity and immunogenicity of ChAd63 CS at two doses, 5×10^9^ virus particles (vp) and 5×10^10^ vp, administered alone and in heterologous prime boost with MVA CS 2×10^8^ plaque forming units (pfu) in healthy malaria-naïve adults (Fig. 1).

### ChAd63 CS and MVA CS Vaccines

Generation of the recombinant vectors has been previously described [21]. They were manufactured under Good Manufacturing Practice conditions by the Clinical Biomanufacturing Facility, University of Oxford (ChAd63 CS) and IDT Biologika, Rosslau, Germany (MVA CS). Previous vectored vaccines expressing the entire CS construct (CS) have been evaluated in Oxford, demonstrating only modest T cell immunogenicity and efficacy on sporozoite challenge [30], [31]. The poor immunogenicity of the standard full length CSP insert used in previous vectors in clinical trials (CSO) [18], [31], [32], suggest that there may be an important difference in the intrinsic immunogenicity of the previously tested CSO insert compared to the ME-TRAP insert. Using information from multiple sources [33]–[35], we have designed a novel CS antigen, to be used in this study, which omits the extreme C-terminus of the protein that encodes the GPI-anchor sequence. The CS insert is a codon-optimised cDNA encoding a C-terminally truncated *Plasmodium falciparum* CS protein. Compared to wild-type, the expressed protein lacks the C-terminal 14 amino acids, in order to inactivate the GPI-anchor signal sequence. It also has a reduced number of NANP repeats. The expression of CS is controlled by a CMV promoter and BGH polyA signal.

Briefly, ChAd63 CS was generated in suspension PC92-GMP cells and purified by caesium chloride density-gradient centrifugation. MVA CS was generated in chicken embryo fibroblasts (CEFs) and purified by sucrose density-gradient centrifugation. Each vaccine lot underwent comprehensive quality control analysis to ensure that the purity, identity and integrity of the virus met pre-defined specifications. Vaccine lots were stored at the clinical site in a temperature-monitored freezer. The immuno-potency of the ChAd CS and MVA CS vaccines was confirmed by immunogenicity evaluation in mice. To ensure on-going stability for both vaccines, MVA CS was tested regularly by titration on CEFs in addition to mouse potency testing and ChAd63 CS was tested by hexon immunostaining for infectious units.

### Participants

The study was conducted at the Clinical Research Centre, Royal College of Surgeons in Ireland, Beaumont Hospital, Dublin, Ireland. Healthy, malaria-naïve males and non-pregnant females aged 18–50 were invited to participate in the study. There was no selection of volunteers on the basis of pre-existing neutralizing antibodies (NAb) to the ChAd63 vector prior to enrolment, (see supporting information for the full list of inclusion and exclusion criteria).

### Study Design

This was a Phase Ia open-label, non-randomized malaria vaccine trial. The clinical trial protocol and supporting CONSORT checklist are available as Supporting Information; see S1 Protocol, S1 Checklist and S1 File. Allocation to study groups (Fig. 1) occurred at screening based on volunteers' availability. Twelve volunteers were vaccinated intramuscularly (IM) with 5×10^9^ vp ChAd63 CS (in 0.9% NaCl and administered in 350 µL) (groups 1A & 1B). Eight of these volunteers were subsequently vaccinated 56 days later with 2×10^8^ pfu MVA CS IM, undiluted and administered in 340 µL (group 1B). Another twelve volunteers were vaccinated IM with 5×10^10^ vp ChAd63 CS undiluted and administered in 350 µL(group 2A & 2B) and eight of these were subsequently vaccinated 56 days with 2×10^8^ pfu MVA CS undiluted and administered in 340 µL. All vaccines were administered in the deltoid region, with volunteers in groups 1B & 2B receiving ChAd63 CS and MVA CS in alternating arms.

Volunteers attended clinical follow-up at days 1, 14, 28, 56 and 90 following ChAd63 CS immunization in groups 1A and 2A and at days 1, 14, 28, 56, 57, 63, 84 and 140 following ChAd63-CS immunization in groups 1B and 2B. Safety assessments, including blood sampling for safety and immunology analysis at these visits were conducted as previously described [29]. Participants were given a diary in which to record adverse events (AEs). A time window ranging between 1 and 14 days was allowed for vaccination and follow-up visits.

### Sample size

This was an observational and descriptive study to assess the safety and immunogenicity of ChAd63 CS and MVA CS. The sample size (*n* = 24) was chosen to allow determination of the magnitude of the primary outcome measures, especially of serious and severe AEs, rather than assessment of statistically significant differences between groups.

### Ethical & Regulatory Approval

The trial VAC038 was registered on the European Medicines Agency database (EudraCT 2011-001875-38). As the trial was initially planned to take place in sites in the UK and Ireland, ethical and regulatory approval was sought from both UK and Irish institutions. Regulatory approval for the study was granted by the UK Medicine and Healthcare Regulatory Authority (MHRA) (ref. 21584/0285/001-000) and by the Irish Medicines Board (CT number CT 900/516/2, Case number 2107330). Ethical approval was granted by the National Research Ethics committee – South Central – Oxford A in the UK (ref. 11/SC/0289) and by the Research Ethics committee of Beaumont Hospital in Ireland (ref. 11/58). Vaccine use was authorized by the Environmental Protection Agency (EPA) of Ireland (Reference number G0451-01) and by the Genetic Modification Committee of the Oxford Radcliffe Hospitals trust (Ref. GM462.11.63). All participants gave written informed consent prior to any study procedure being undertaken. The study was conducted according to the principles of the Declaration of Helsinki (2008) and the International Conference on Harmonization (ICH) Good Clinical Practice (GCP) guidelines. The Local Safety Committee provided safety oversight and GCP compliance was independently monitored by an external organization (Appledown Clinical Research Ltd, Great Missenden, UK).

### Safety

The first volunteer to receive each vaccine at each dose was vaccinated alone and observed in clinic for 12 hours. They were then reviewed again in clinic 24 hours post vaccination. Once 72 hours had elapsed and in the absence of safety concerns, volunteers 2 and 3 were vaccinated with that vaccine and dose. Once 72 hours had elapsed, and in the absence of safety concerns, other volunteers were administered the vaccine at the same dose. Apart from the first volunteer to receive each vaccine at a particular dose, all volunteers were observed in clinic for 30 minutes after each immunization. Prior to dose escalation of the ChAd63 CS vaccine from 5×10^9^ vp to 5×10^10^ vp, the independent data safety monitoring board reviewed and approved a report of all safety data collected from volunteers up to 14 days after receiving 5×10^9^ vp ChAd63 CS.

Volunteers were given a digital thermometer, injection site reaction measurement tool and symptom diary card to record their daily temperature, injection site reactions and solicited systemic AEs for 14 days following vaccination with ChAd63 CS and 7 days following vaccination with MVA CS. Local and systemic reactogenicity was evaluated at subsequent clinic visits and graded for severity, outcome and association to vaccination as per the criteria outlined in S1, S2 and S3 Tables. Blood was sampled at all visits post vaccination except days 1 and 57, and the full blood count with differential, platelet count and serum biochemistry (including electrolytes, urea, creatinine, bilirubin, alanine aminotransferase, alkaline phosphatase and albumin) measured.

### Peripheral Blood Mononuclear Cell (PBMC) and Serum Preparation

Blood samples were collected into lithium heparin-treated Vacutainer blood collection systems (Becton Dickinson, UK). PBMC were isolated and used within 6 hours in fresh assays as previously described [29]. Excess cells were frozen in foetal calf serum (FCS) containing 10% dimethyl sulfoxide (DMSO) and stored in liquid nitrogen. For serum preparation, untreated blood samples were stored at 4°C and then the clotted blood was centrifuged for 5 min (1000 *xg*). Serum was stored at −80°C.

### Peptides for T cell Assays

Peptides (NEO Peptide, Cambridge, MA, USA), 15 amino acids (aa) in length and overlapping by 10 aa spanning the entire CSP insert, were reconstituted in 100% DMSO at 50–200 mg/mL and combined into various pools for ELISPOT and flow cytometry assays. The composition of peptide pools containing 2 to 15 peptides are listed in S5 Table CSP overlapping peptides.

### *Ex-vivo* interferon-γ (IFN-γ) ELISPOT

The kinetics and magnitude of the T cell response to CSP were assessed over time by *ex-vivo* IFN-γ ELISPOT following an 18–20 hour re-stimulation of PBMC with overlapping peptides spanning the entire CSP insert present in the viral vectored vaccines (S5 Table peptides). Fresh PBMC were used in all ELISPOT assays using a previously described protocol [29], except that CSP peptide pools (final concentration each peptide 5 µg/mL) were added to test wells, culture medium was added to negative un-stimulated wells, and Staphylococcal enterotoxin B (SEB, Sigma) (final concentration 10 µg/mL) plus phytohemagglutinin (PHA, Sigma) (final concentration 0.02 µg/mL), PPD (10 µg/mL) and FEC (pool of peptides from influenza, Epstein Barr virus and cytomegalovirus, final concentration 10 µg/mL, Neo peptide) was added to positive control wells [36]. Each well contained 200,000 PBMC. Spots were counted using an ELISPOT counter (Autoimmun Diagnostika (AID), Germany). Results are expressed as IFN-γ spot-forming cells (SFC) per million PBMC. Background responses in un-stimulated control wells were almost always less than 20 spots, and were subtracted from those measured in peptide-stimulated wells. Responses are shown as the summed response to all the CSP peptide pools (unless otherwise stated). 10% of all ELISPOT plates underwent a quality control (QC) procedure involving review of time-lines of cell processing, visual inspection of plate, review of raw data output and validation of positive and negative controls. Positive controls were deemed valid if there were at least 200 spots in one of the positive control wells (PHA, SEB or FEC). Two negative control wells containing only medium and PBMC where required on each plate. In order to pass QC the mean count of these two well had to be less than 20 spots per well.

### Multiparameter Flow Cytometry

Responses were assessed by 7 colour flow cytometry, which was performed on frozen PBMC, by stimulating aliquots of 1×10^6^ cells in 1 ml of medium containing anti-CD28 and anti-CD49d at 1 µg/ml (Becton Dickinson) and 5 µl/ml of CD107a-PeCy5 (eBioscience) with either no antigen, a pool of all 55 peptides spanning the T9/96 the CSP antigen (2 µg/ml) or a positive control, Staphylococcal enterotoxin B (Sigma, 1 µg/ml), in 5 ml polystyrene FACS tubes for 18 hours. Brefeldin A and Monensin, both at 1 µg/ml, were added for the last 16 hours. Cells were incubated with a dead cell discrimination dye (VIVID, 1/80, Invitrogen), and then surface stained at 4°C with CD4-APC (1/20, eBioscience) or CD4-Qdot 625 (1/50, Invitrogen), CD14- and CD19-Pacific Blue (both 1/50, Becton Dickinson). After permeabilisation, intracellular staining was performed at room temperature with CD3-PeCy5 (part A, 1/20, eBioscience) or CD3-Alexa Fluor 700 (part B, 1/100, eBioscience) plus CD8-APC-Alexa Fluor 780 (1/50) and IFN-γ-FITC (1/50), IL-2-PE (1/100) and TNFα-Pe-Cy7 (1/50, all eBioscience) and fixed in 1% paraformaldehyde. Acquisition was performed on the day of staining on a BD LSRII; at least 500,000 events were collected per sample. Data was prepared and analysis performed using FlowJo v8.8.6 (Treestar Inc,), Pestle v1.6 and Spice v5.05 (Mario Roederer, Vaccine Research Centre, NIAID, NIH). Dead cells (Vivid^+^), monocytes (CD14^+^), and B cells (CD19^+^) were excluded from the analysis. A time gate was first evaluated, and then cells were gated on lymphocytes, singlets, live CD3^+^, CD8^+^ or CD4^+^ (excluding double-positives), and then IFNγ and combinations of markers. A sample gating strategy is provided in S1 Figure. Responses were determined after subtraction of the response in the unstimulated control for each sample. Pie charts were created using absolute measures with a threshold of 0.001%. MFI (Mean Fluorescence Integrity) was calculated using the geometric mean of the cytokine-positive population and iMFI (integrated MFI) represents the integration of the frequency with the geometric mean of the cytokine-secreting population, giving a measure of total amount of cytokine production.

### Total IgG ELISA and Human IFA

Anti-CSP antibodies were measured at Walter Reed Army Institute of Research, by enzyme-linked immunosorbent assay (ELISA) against the CSP repeat region using a hexameric synthetic peptide (NANP)_6_ (CSPrp ((NANP)_6_ Peptide [100 µg/mL] Eurogentec Cat: EP070034 Lot: 14) and immunofluorescent antibody assay (IFA) using air-dried sporozoites. (S2 File for details of assay).

### Statistical Analysis

Data were analyzed using GraphPad Prism version 5.04 for Windows (GraphPad Software Inc., California, USA). Geometric mean or median responses for each group are described. Kruskall-Wallis test was used for one-way analysis of variance by ranks. Significance testing of differences between two groups used the two-tailed Mann-Whitney U test or Wilcoxon signed rank test as appropriate. No corrections for multiple hypothesis testing were used.

## Results

### Study Recruitment

Recruitment took place between December 2011 and July 2012. Twenty-four healthy malaria-naïve adult volunteers (12 female and 12 male) were enrolled, immunized and followed up (Fig. 1). The mean age of volunteers was 30 years (range 21–46). Full demographic information on volunteers is available in S6 Table. Vaccinations began in January 2012 and all follow-up visits were completed by November 2012. All volunteers attended all visits as scheduled and completed the study.

### Safety and Reactogenicity

No unexpected or serious AEs occurred and no volunteers were withdrawn due to AEs.

**Figure 2:**
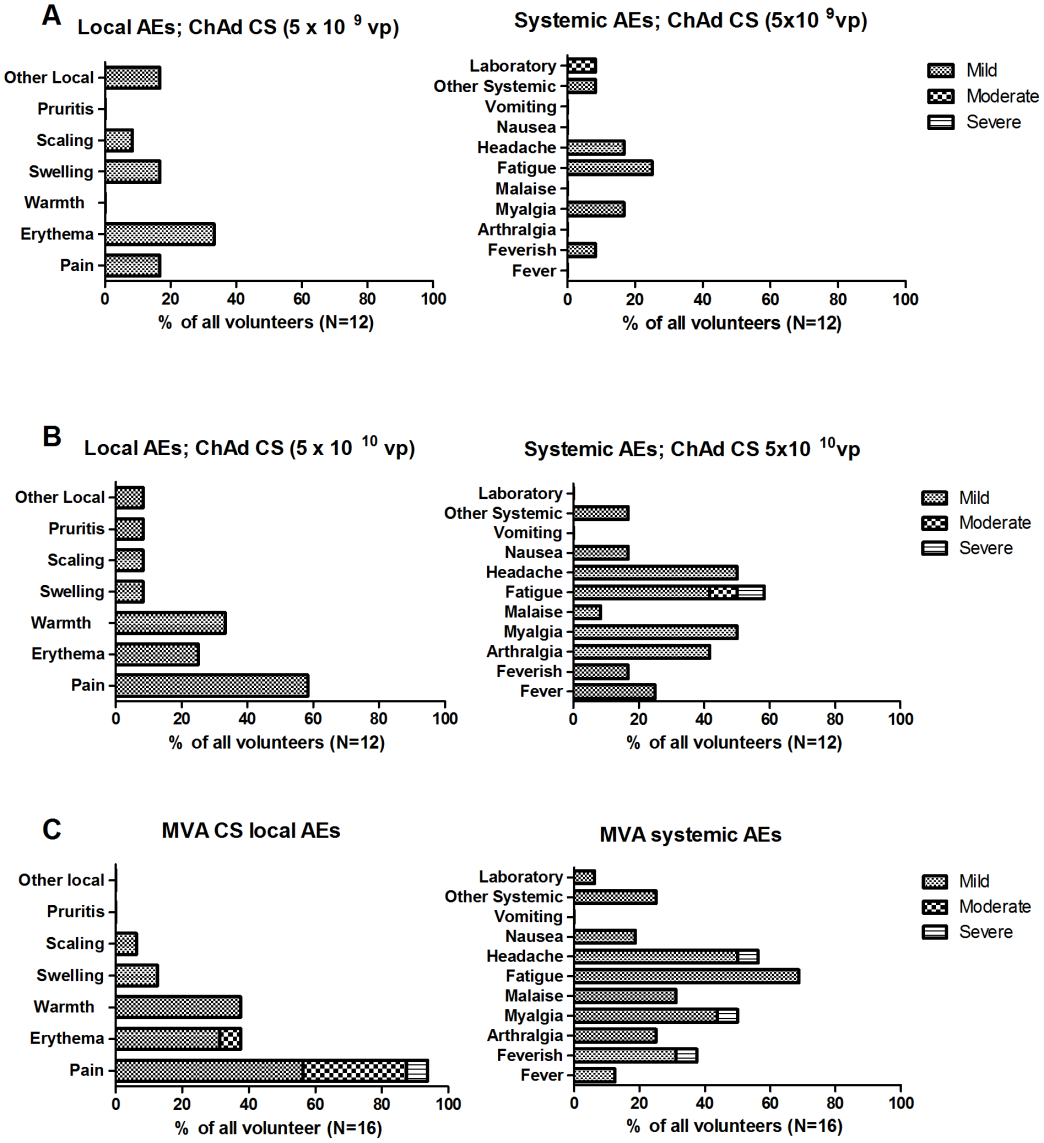
Local and systemic adverse events (AEs) possibly, probably or definitely related to vaccination shown as percentage of volunteers affected. (a) Post ChAd63 CS 5×10^9^ vp; (b) Post ChAd63 CS 5×10^10^ vp; (c) Post MVA CS 2×10^8^ pfu.

**Figure 3:**
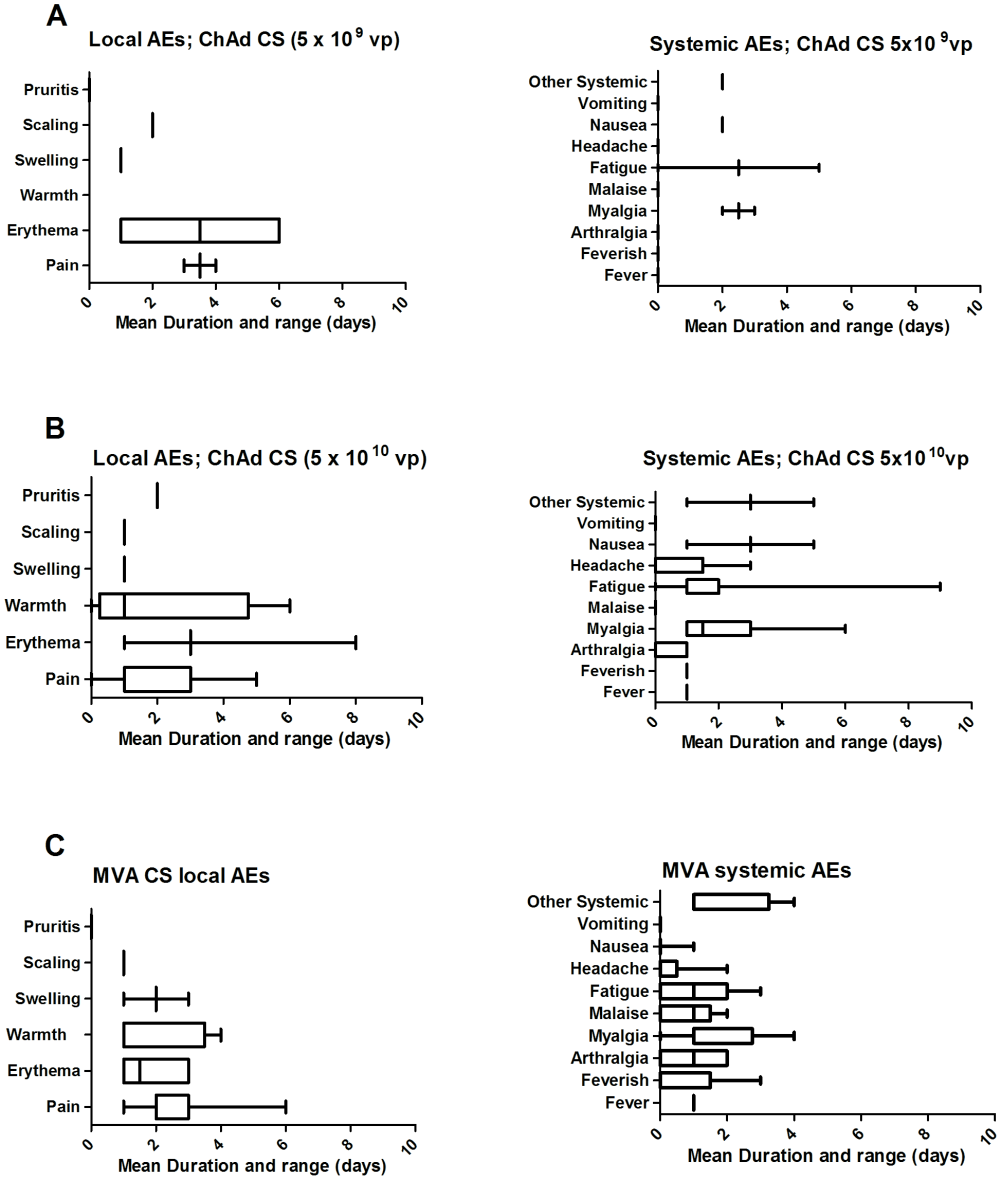
The mean duration and range of duration of local and systemic AEs possibly, probably or definitely related to vaccination. (A) post ChAd63 CS 5×10^9^ vp; (B) post ChAd63CS 5×10^10^ vp; (C) post MVA CS.

Table 1 provides details of AEs deemed possibly, probably or definitely related to vaccination. ChAd63 CS demonstrated a good safety profile with the majority of AEs being mild in severity (91%) and 80% of all AEs resolved within 48 hours (Figs. 2 & 3). Overall, 14 out of 24 volunteers (58%) experienced one or more local AEs related to ChAd63 CS; all of which were mild. 20 out of 24 volunteers (83%) experienced one or more systemic AE related to ChAd63 CS and a dose response was seen for systemic reactogenicity, with a greater proportion of volunteers receiving 5×10^10^ vp experiencing a systemic AE than volunteers receiving 5×10^9^ vp. The majority of these AEs were mild in severity. MVA CS administered 8 weeks after the ChAd63 CS was more reactogenic, with 14 out of 16 volunteers (87%) experiencing at least one local AEs, mainly pain, erythema and warmth. 15 volunteers (93%) experienced at least one systemic AEs, including feverishness, myalgia, fatigue, malaise and headache in the 24 hours following vaccination, though the majority of these were mild in severity. This AE profile is similar to the flu-like symptoms that have been reported in the past with similar doses of MVA vectored vaccines expressing other antigens [20], [22], [29]–[32], [37]–[40]. The neutrophil count of one volunteer dropped from 1.56×10^9^/L at screening to 1.09×10^9^/L 28 days post vaccination with ChAd63 CS at a dose of 5×10^9^ vp and remained less than 1.56×10^9^/L throughout follow up.

**Table 1:** Local, systemic and laboratory adverse events post immunization.

Sign or Symptom	ChAd63 CS 5×10^9^			ChAd63 CS 5×10^10^			MVA CS		
	N = 12			N = 12			N = 16		
	(% of volunteers)			(% of volunteers)			(% of volunteers)		
	Mild	Moderate	Severe	Mild	Moderate	Severe	Mild	Moderate	Severe
**LOCAL**									
**Pain**	2(17%)	0	0	7(58%)	0	0	9(56%)	5(31%)	1(6%)
**Erythema**	4(33%)	0	0	3(25%)	0	0	5(31%)	1(6%)	0
**Warmth**	0	0	0	4(33%)	0	0	6(37%)	0	0
**Swelling**	2(17%)	0	0	1(8%)	0	0	2(12%)	0	0
**Scaling**	1(8%)	0	0	1(8%)	0	0	1(6%)	0	0
**Pruritis**	0	0	0	1(8%)	0	0	0	0	0
**Other**	2(17%)	0	0	1(8%)	0	0	0	0	0
**Total local AEs**	11	0	0	18	0	0	23	6	1
**SYSTEMIC**									
**Fever**	0	0	0	3(25%)	0	0	2(12%)	0	0
**Feverish**	1(8%)	0	0	2(17%)	0	0	5(31%)	0	1(6%)**
**Arthralgia**	0	0	0	5(42%)	0	0	4(25%)	0	0
**Myalgia**	2(17%)	0	0	6(50%)	0	0	7(44%)	0	1(6%)**
**Malaise**	0	0	0	1(8%)	0	0	5(31%)	0	0
**Fatigue**	3(25%)	0	0	5(42%)	1(8%)	1(8%)	10(62%)	0	0
**Headache**	2(17%)	0	0	6(50%)	0	0	8(50%)	0	1(6%)**
**Nausea**	0	0	0	2(17%)	0	0	3(19%)	0	0
**Vomiting**	0	0	0	0	0	0	0	0	0
**Other systemic**	1(8%)	0	0	2(17%)	0	0	4(25%)	0	0
**Laboratory**	1(8%)*	0	0	0	0	0	1(6%)*	0	0
**Total systemic AEs**	10	0	0	32	1	1	49	0	3

Adverse events deemed possibly, probably or definitely related to vaccination are shown. ‘Other systemic’ following MVA CS included nasal congestion, laryngitis and pharyngitis. The highest intensity adverse event per subject is listed. Other local AEs included paraesthesia. All ‘other’ AEs were considered possibly related to vaccination due to a temporal association.

*The two laboratory AEs related to the same volunteer, who experienced neutropenia following both vaccination.

**Three severe systemic AEs followed MVA CS, all experienced by one volunteer simultaneously and resolved within 48 hours of vaccination.

### ChAd63-MVA CS T cell immunogenicity assessed by *ex-vivo* IFN-γ ELISPOT

**Figure 4:**
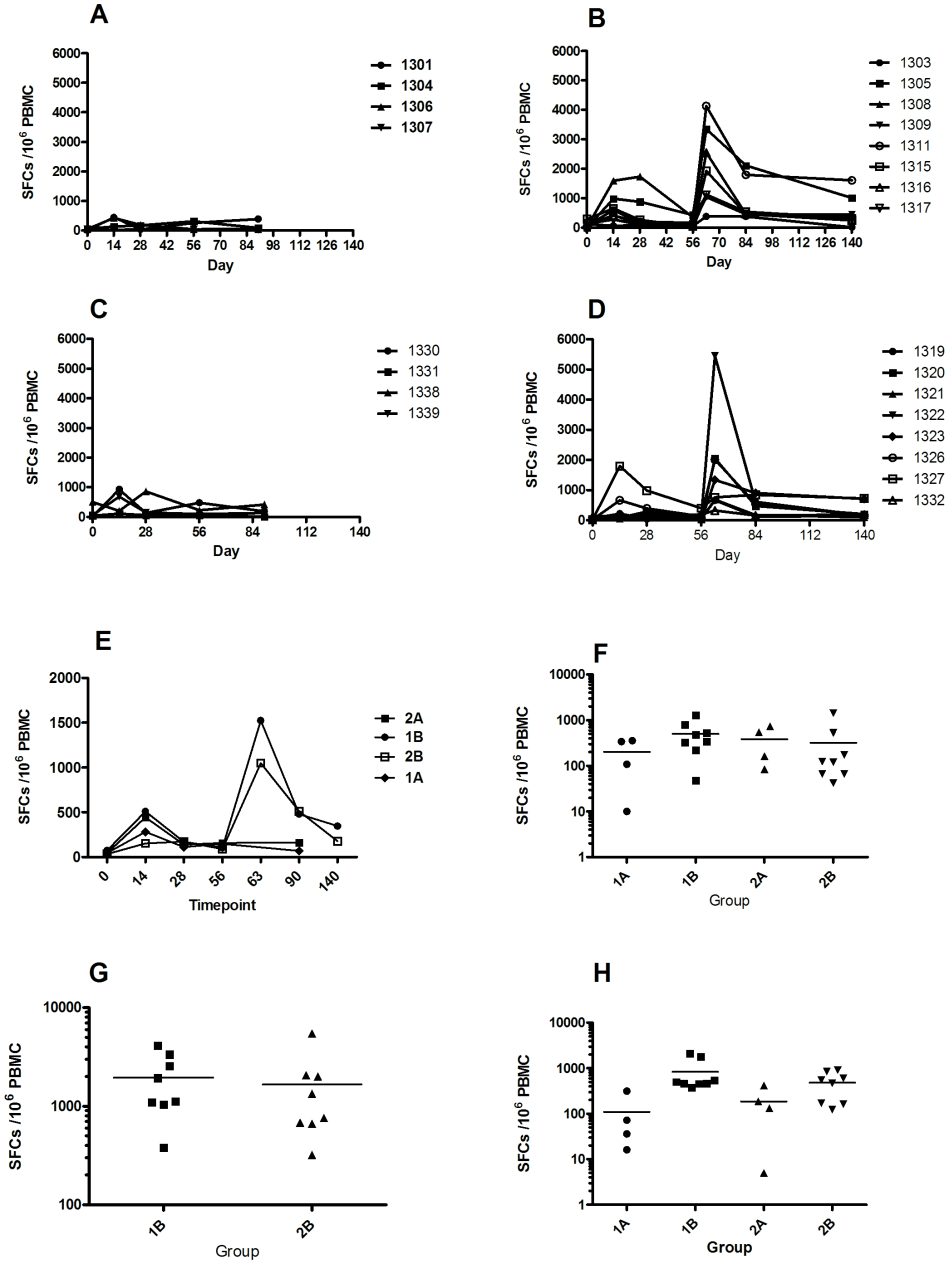
Summary of PBMC IFN-y ELIspot responses of volunteers in each group. Summed SFC/million PBMCs. (A) and (B) individual responses for groups 1A and 1B respectively over time. (C) and (D) show individual responses for groups 2A and 2B respectively over time. (E) median ELIspot response by group by time-point, changes were significant over time; for group 1B, day 0 to 14 p = 0.0063; day 14 to 63 p = 0.0148; day 0 to 63 p = 0.0002, Mann-Whitney test; and group 2B, day 0 to 14 p = 0.01; day 14 to 63 p = 0.0074; day 0 to 63 p = 0.0002, Mann-Whitney test. (F), (G) & (H) show individual responses by group at days 14, 63 and 84 or 90 respectively.

Antigen-specific T cell responses in all volunteers as measured by *ex-vivo* IFN-γ ELISPOT are shown in Fig. 4. When comparing the responses to two different doses of ChAd63 CS, no significant difference was seen between group 1 (ChAd63 5×10^9^ vp) and group 2 (ChAd63 CS 5×10^10^ vp) at the peak of the response on day 14 (median 423 [range 12.5–1590] vs 178 [range 52.5–1795] SFC/million PBMCs, p = 0.54 Mann-Whitney test). Thereafter T cell responses gradually contracted to day 56 (Fig. 4). Administration of MVA CS at day 56 significantly boosted responses in all volunteers as measured 7 days later on day 63 (Fig. 4). No significant difference was seen when comparing day 63 ELIspot responses between groups 1B & 2B (median 1523, [range 380–4125] vs 1048 [range 320–5450] SFC/million PBMCs in groups 1B and 2B respectively, n = 8 v 8, p = 0.5 Mann-Whitney test). ELIspot responses subsequently contracted but remained above baseline when measured at the last time-point, day 140, (median 1B 346.25 [range 17.5–1607], median 2B 176 [range 102–707] SFC/million PBMCs), Fig. 4. There was no significant difference between the groups at this time-point (p = 0.16 Mann-Whitney test). Median ELISPOT response in groups 1B and 2B were significantly different when all time-points were analysed (p<0.0001 for both groups, Kruskall-Wallis). The changes were significant over time; group 1B, day 0 to 14 p = 0.0063; day 14 to 63 p = 0.0148; day 0 to 63 p = 0.0002, Mann-Whitney test; group 2B, day 0 to 14 p = 0.01; day 14 to 63 p = 0.0074; day 0 to 63 p = 0.0002, Mann-Whitney test. This was not seen with groups 1A and 2A, p = 0.66 and p = 0.014 respectively, Kruskall-Wallis.

### Breadth of the CSP T cell response

**Figure 5:**
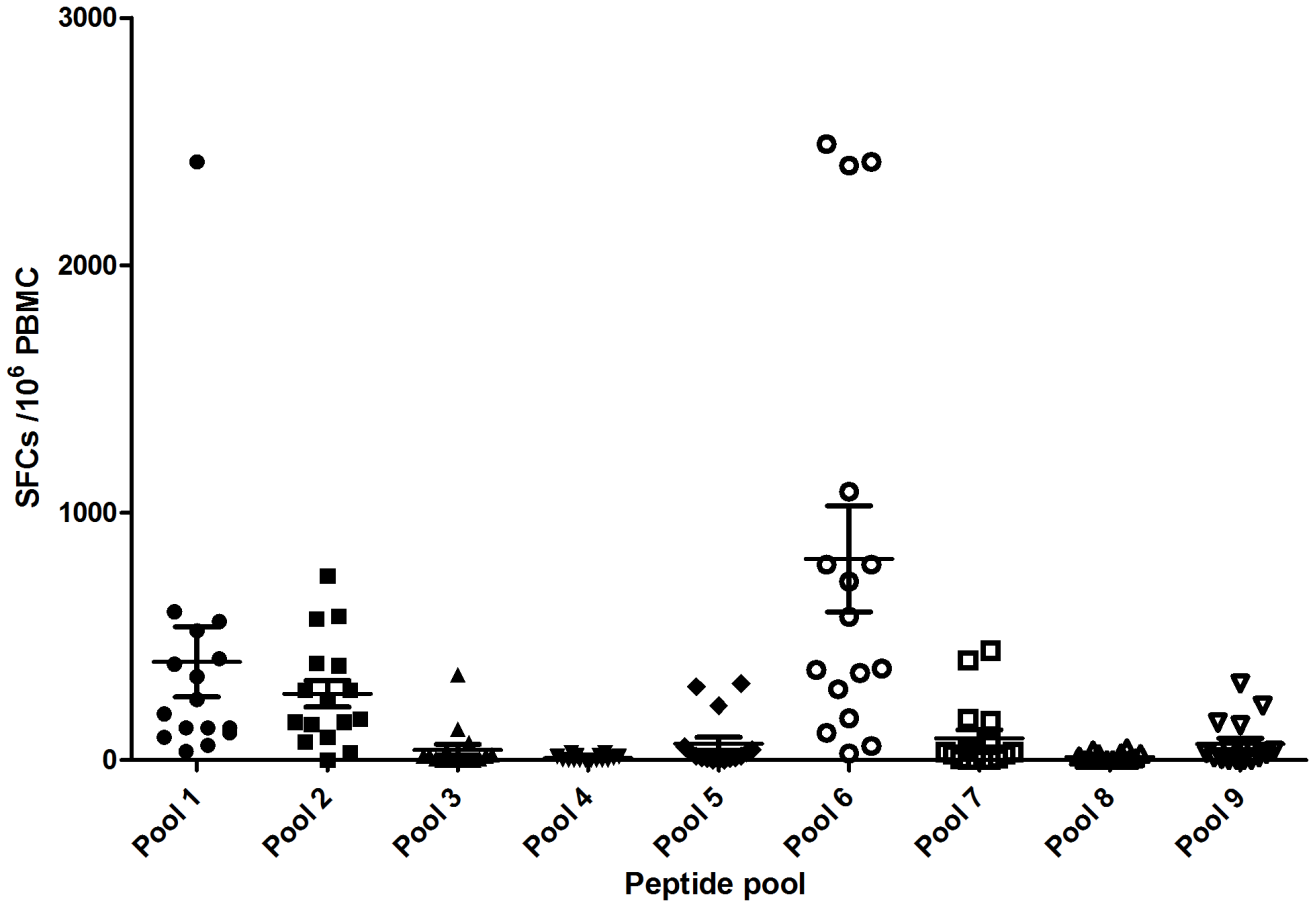
Individual ELISpot responses in SFC/10^6^ PBMC at day 63 by peptide pool. Bar represents mean, whiskers; standard error of the mean.

T cell responses in all volunteers were detected in multiple peptide pools spanning the entire CSP vaccine insert in the *ex-vivo* IFN-γ ELISPOT assay, (Fig. 5).

Following priming immunization with ChAd63 CS individual responses were seen across all pools with no apparent immune-dominant region in CS detected. One week post boost with MVA CS, responses were again observed across all pools. Pools 1, 2, 5, 6 showed a significant increase in ELISPOT response when comparing pre and post boost results, with p values of 0.0229, 0.0021, 0.0227, 0.0026 respectively (Wilcoxon matched pairs signed rank test).

**Figure 6:**
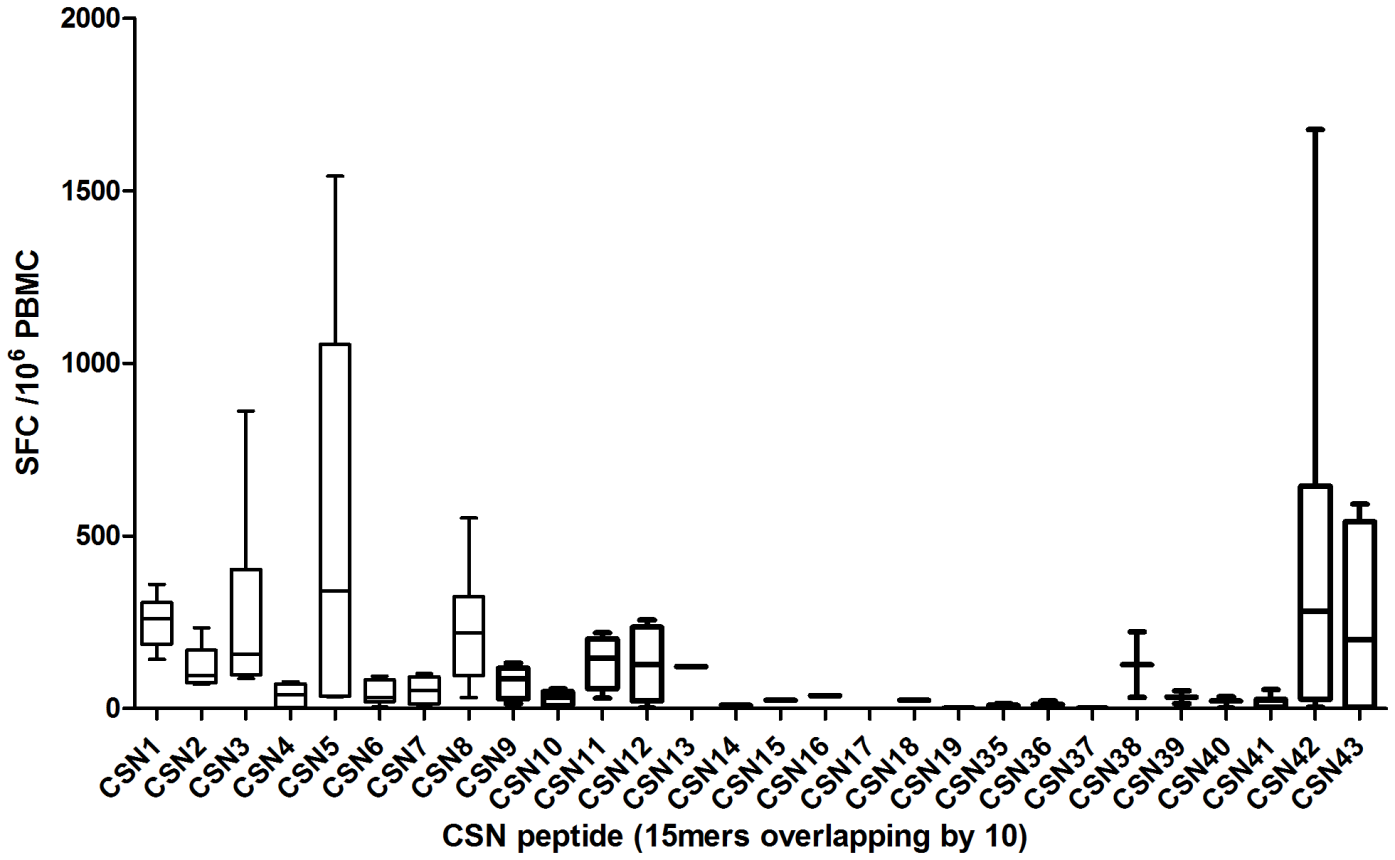
Median & Interquartile range ELISpot responses in SFC/10^6^ PBMC at day 63 by peptide. Box plots of the medians and 25% and 75% percentiles of response to each peptide. The first and third quartiles are the top and base of each box, the upper and lower bars represent the maximum and minimum values respectively.

The 6 highest responders at day 63 were assessed by ex-vivo IFN-γ ELISPOT for responses to single peptides in an attempt to map the most immunogenic epitopes of the CS insert. Median responses to peptides 1 to 43 (15 mer peptides overlapping by 10aa) are shown in Fig. 6. Peptides 1, 3, 5, 8, 42 and 43 were identified as containing dominant epitopes, although it is likely that there is a single epitope spanning peptides 42 and 43.

### CSP T cell multi-functionality

**Figure 7:**
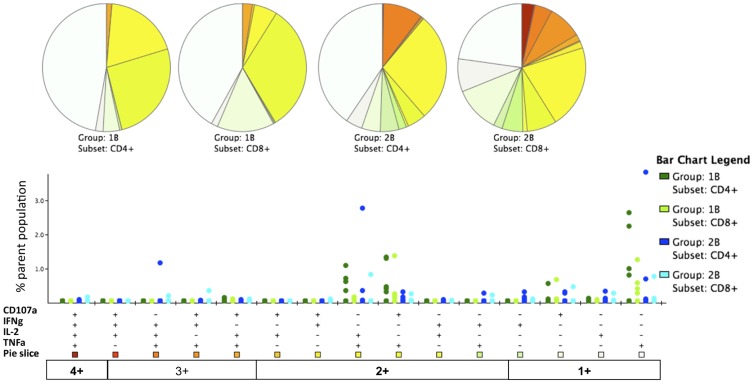
T cell multi-functionality following ChAd63-MVA CS immunization. The multi-functionality of the CD4^+^ and CD8^+^ T cell responses was assessed by polychromatic flow cytometry and ICS. Frozen PBMCs from day 63 were re-stimulated with a pool of CSP peptides and cells stained as described in methods above. Gating strategy and representative plots are shown in S1 Figure. Responses are grouped and colour-coded according to the CD4^+^ and CD8^+^ subsets, and the number of functions detected for each T cell population. Individual data points showing the percentage of the parent CD4^+^ or CD8^+^ response are shown for each of the functional populations indicated on the X-axis. The pie charts summarize the fractions of CSP specific CD4^+^ or CD8^+^ T cells that are positive for a given number of functions (CD107a, IFNγ, IL-2 and TNFα). The row at the base of the figure labelled ‘pie chart’ provides a key to the colours of pie segments, the darkest colours representing cells that produced 4 cytokines and the lightest colour producing one.

**Figure 8:**
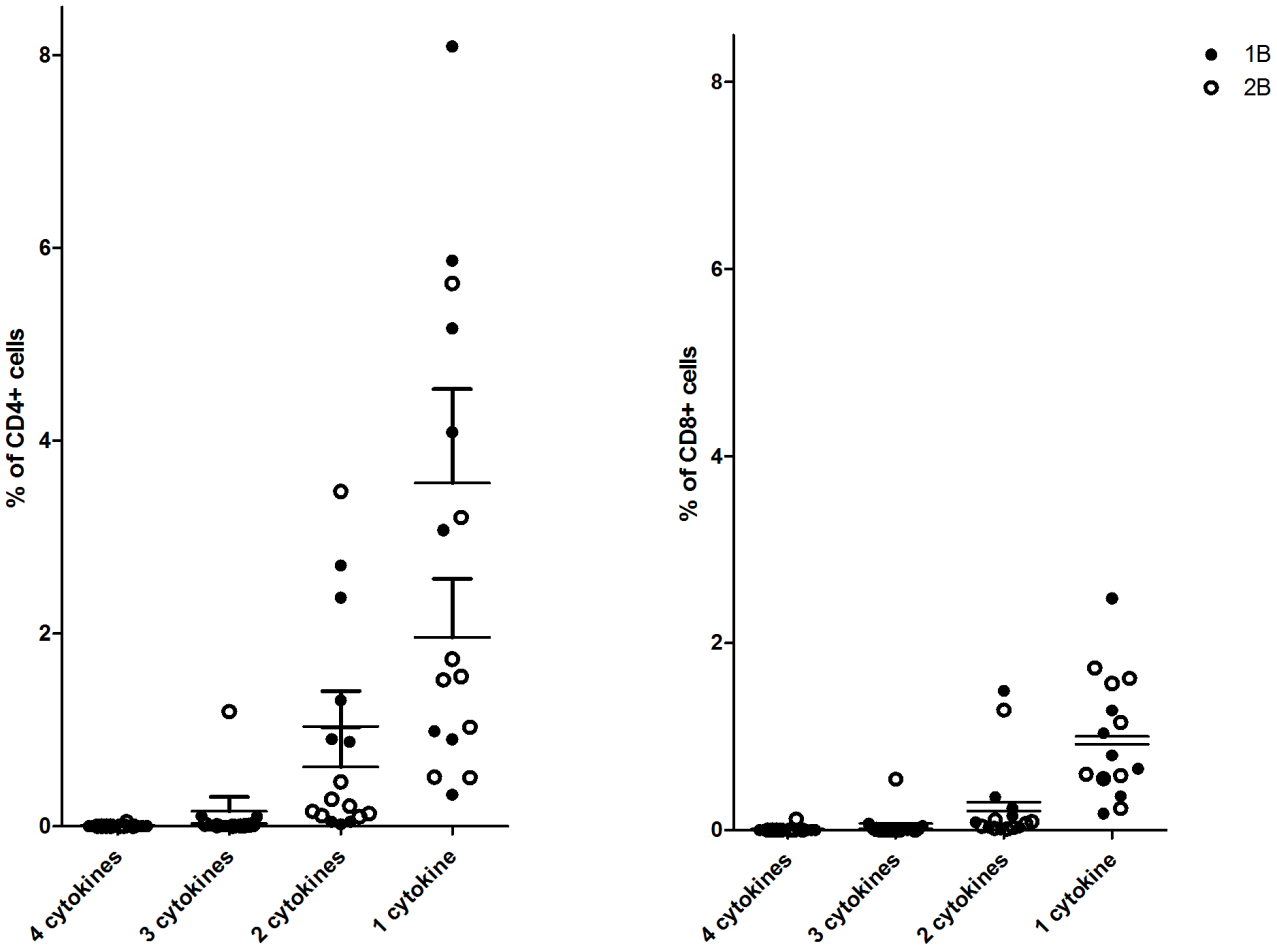
Multifunctional antigen specific cells. Percent of CD4^+^ and CD8^+^ PBMCs at day 63 producing 1–4 cytokines following stimulation with CS peptide pools.

Antigen-specific CD3^+^ T cell functionality was also assayed by ICS at the days 14, 63 and 84/90 time-points. Following peptide re-stimulation, detectable CSP-specific CD3^+^ T cells consisted of a mixed CD4^+^ and CD8^+^ phenotype, Fig. 7. It should be noted that the ELISpot and intracellular cytokine staining (ICS) assays vary in methodology (including the use of multiple versus a single peptide pool respectively, differences in peptide concentration, use of co-stimulatory antibodies and the use of fresh versus frozen PBMC). As no difference in ELIspot response was seen between volunteers receiving different doses of ChAd63 CS, data for ICS was combined across groups 1B & 2B and assays were performed where cell numbers allowed. Fig. 8 presents the relative proportion of multifunctional cells at the peak observed at day 63, it does not provide detail on which cytokine is being produced.

**Figure 9:**
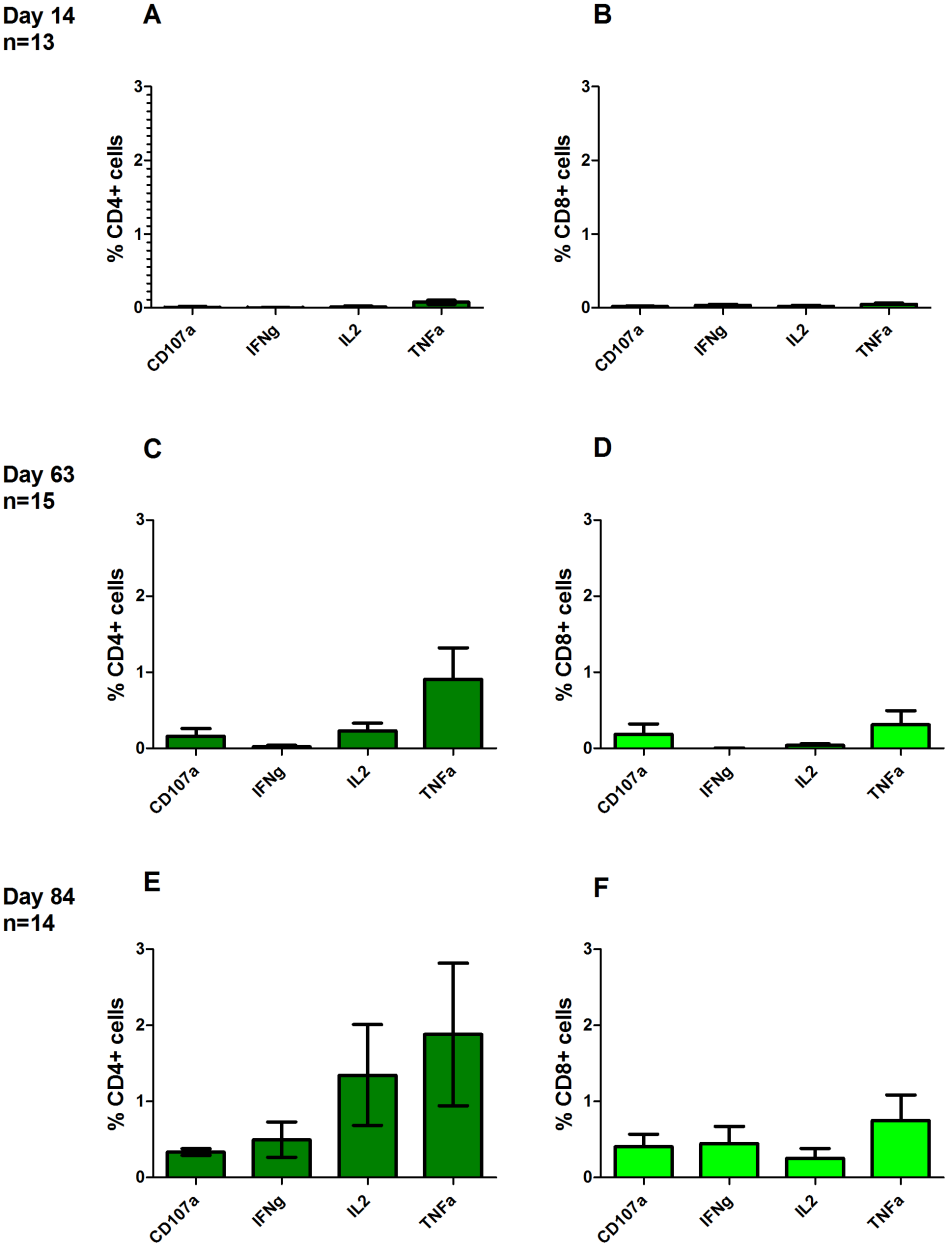
Cytokine production by cell type and time-point assessed by 7 colour flow cytometry. Mean percent and standard error of the mean (SEM) of CD4+ and CD8+ PBMCs producing antigen-specific cytokines at given time-point post vaccination are shown for each cytokine. (A) percent CD4+ and (B) percent CD8+ PBMCs producing CD107a, IFNg, IL2 and TNFa at day 63. (C) percent CD4+ and (D) percent CD8+ PBMCs producing CD107a, IFNg, IL2 and TNFa at day 63. (E) percent CD4+ and (F) percent CD8+ PBMCs producing CD107a, IFNg, IL2 and TNFa at day 84.

Across all three time-points analysed CD107a (marker of degranulation) expression was up-regulated by both CD4^+^ and CD8^+^ T cells. Fig. 9. CD4^+^ cells produced higher levels of TNFα than CD8^+^ cells at all time-points, but this was not statistically significant (*p* = 0.58, *p* = 0.31 and *p* = 0.48 for days 14, 63 and 84 respectively, Mann-Whitney test). CD4^+^ cells also produced greater levels of IL-2 compared to CD8^+^ cells at all time-points, however this did not reach significance (*p* = 0.77, *P* = 0.59 and *p* = 0.32 for days 14, 63 and 84 respectively, Mann-Whitney test). Negligible levels of IFN-γ were produced by either CD4+ or CD8+ cells at days 14 and 63. Levels comparable to IL-2 and TNFα were observed at day 84. Distinct populations of CD4^+^ and CD8^+^ T cells expressing 1+, 2+, 3+ or 4+ functional markers/cytokines were evident following a Boolean gate analysis (Fig. 7 and S7 Table).

### ChAd63-MVA CS antibody immunogenicity assessed by ELISA and Immuno-fluorescence assay (IFA)

**Figure 10:**
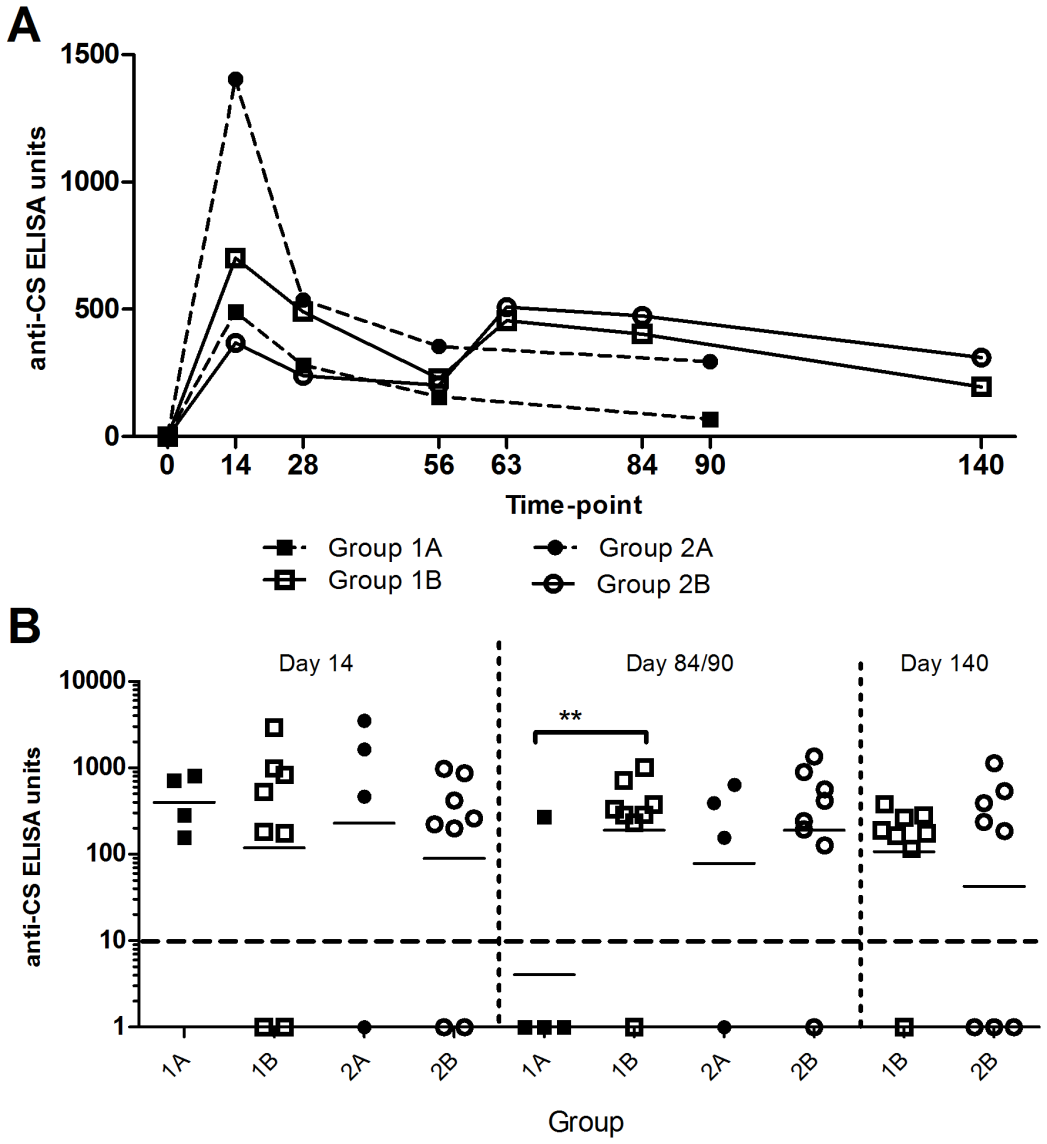
Summary IgG antibody titre against CS. (A) Mean serum IgG ELISA antibody response against CS over time in days post vaccination are shown. (B) individual and geomean antibody responses by group and time point. ** p<0.05 by Mann-Whitney test.

The kinetics and magnitude of the serum IgG antibody response against CS were assessed over time by ELISA (Fig. 10). All volunteers had IgG titres below the limit of detection at day zero. CS-specific IgG was induced in all volunteers. Mean responses peaked at day 14 with CS antibody titre of 631 and 713 for groups 1(A&B) and 2(A&B) respectively. Boosting with MVA CS resulted in a significant increase in antibody concentration in group 1B, compared to the un-boosted group 1A, as measured at day 84/90 (*p* = 0.037; Mann-Whitney test). However this was not seen when comparing groups 2A and 2B at the same time-points (*p* = 0.49 Mann-Whitney test). Mean antibody response was higher in group 2B compared to group 1B at day 140, but this difference was not statistically significant (*p* = 0.87 Mann-Whitney test). Samples from any volunteer with ELISA titres above the Lower limit of detection (LLD) at both day 28 and 84/90 post ChAd63 CS, were also assessed by IFA. No significant boosting effect was observed in either group 1B and 2B when analysing by IFA, p = 0.08 (Mann-Whitney test).

### HLA typing and immune response

Previous studies have identified class I and II CSP epitopes and their HLA restriction, [41]–[43] so all volunteers were typed for major HLA types; A, B and DR (S4 Table). The apparent immune-dominance of peptide pools 1 and 6 may be explained by the fact that HLA super types A1 and A2, predominate in our volunteers and that it has previously been shown that epitopes contained within these pools are restricted to these HLA super types.

## Discussion

Here we have shown in a first in human phase Ia study, that ChAd63-CS and MVA–CS have acceptable safety profiles, and are potently immunogenic, inducing high levels of antigen-specific multifunctional CD4+ and CD8+ T lymphocytes, and significant levels of antibody.

### Safety

No serious adverse events occurred during the course of the trial. The majority of AEs observed were mild in intensity and resolved rapidly. Over 300 healthy volunteers have now received ChAd63 encoding the malaria antigens ME-TRAP, MSP1 and AMA1 [18], [20]–[22], [31], [44]. The safety profile seen with ChAd63 CS was very similar to that of other ChAd63 vectored vaccines. [20]–[22], [29] MVA CS at a dose of 2×10^8^ was considerably less reactogenic than was observed at higher or same dose of MVA expressing different antigens [20] [22], [29]. 1−2×10^8^ pfu has consistently been shown to be the optimal dose of MVA [20], [22], [24], [28], [30], [32], [38]–[40], [44]. The majority of volunteers who received MVA CS experienced a range of symptoms comprising of feverishness, fatigue, headache and myalgia. These were mild in severity in the majority of cases and all resolved within 48 hours. Comparing ChAd63 CS to NMRC-M3V-Ad-PfC, the other viral vectored CS vaccine which has been studied in humans, both result in mainly mild local and systemic AEs. ChAd63 CS at both doses resulted in less local pain than NMRC-M3V-Ad-PfC; with 17%, 58%, 67% and 86% of volunteers experiencing injection site pain following ChAd CS(5×10^9^ vp), ChAd CS(5×10^10^vp) and NMRC-M3V-AdPfC, first immunization and second immunization, respectively [41]. ChAd63 CS, at either dose, resulted in local erythema in a higher percentage of volunteers, 33%(5×10^9^vp) and 25%(5×10^10^vp) than NMRC-M3V-Ad-PfC, 0% [41]. ChAd63 CS at the higher dose (5×10^10^vp) was also more reactogenic in terms of the systemic AEs of myalgia, headache and fatigue, than NMRC-M3V-Ad-PfC, but the majority were of mild severity and resolved within 48 hours [41]. The safety data collected in this study adds to the already significant body of data supporting the excellent safety profile of this vaccine delivery platform.

### Immunogenicity

MVA expressing CS has been studied in humans in the past and IFN γ ELIspot results have varied from a mean of 79 SFC/10^6^ PBMC when combined with DNA prime [18], to 250 SFC/10^6^ PBMC, when primed with RTS,S, to 1000 SFC/10^6^ PBMC when primed by attenuated fowl-pox virus (FP9) [45]. ChAd63–MVA vectored vaccines have consistently yielded high levels of T-cells [20]–[22], [24], [28], [29], [44]. Despite the fact that CS was one of the earliest recognised target antigens in the development of a malaria vaccine, it remains a leading antigen for vaccine development. Others have also recently published data on a human adenovirus vectored vaccine expressing CS (NMRC-M3V-Ad-PfCA), which yielded a peak mean IFN γ ELISpot CS of 422 SFC/million [41], [42]. ChAd63 –MVA CS yielded a peak mean three fold greater at 1523 SFC/million. Protection assessed by controlled human malaria infection was disappointing in the case of NMRC-M3V-Ad-PfCA, with 2/11 volunteers showing delayed onset of parasitaemia with no volunteer protected [41]. Peak antibody responses to CS for both vaccines measured by ELISA were modest, with NMRC-M3V-Ad-PfCA yielding median ELISA titres of 300 [41], 692 [42] and Ch63-MVA CS yielding a median titre of 631. RTS,S is the malaria vaccine most advanced in clinical development and is currently undergoing phase 3 trials [46]. It is formed from the fusion of CS to the surface antigen of hepatitis B virus to form virus-like particles. Analysis of the immunological correlates of immunity induced by the RTS,S/AS01 vaccine and adjuvant suggest that very high levels of antibodies to CS correlate with protection in humans [47], [48]. However, this correlation is relatively weak and there might be a component of T cell mediated protection induced by the vaccine, even though the magnitude of the T cell response measured after vaccination is modest (mean of approximately 150 SFU/million PMBCs on ELIspot) [49], [50]. The prime-boost strategy of the viral vectored vaccines, ChAd63 and MVA both expressing CS presented here, has produced T cells responses much greater (mean 1,947 SFC/million PMBCs on ELIspot) than RTS,S. The production of CD8+ monofunctional cytokine producing cells which has been shown here (Fig. 8), has been correlated with protection to controlled human malaria challenge in the past [28]. The breadth of the ELISPOT response observed may also be relevant to efficacy. The greater magnitude of T-cell immunogenicity induced by ChAd-MVA heterologous prime-boost immunization correlates with an increase in the number of detectable epitopes recognized [21], [49], [51] so it is likely that increased breadth also correlates with efficacy.

CSP specific antibody responses seen with ChAd63 CS followed by MVA CS were modest (mean of 1.9 µg/mL for groups 1B and 2B 7 days post MVA CS). In contrast RTS,S has yielded mean antibody responses of 78 µg/mL in non-immune adult vaccinees [51]. The significant levels of T cells yielded by vaccination with ChAd63 MVA CS and the high antibody levels produced by RTS,S, raises the possibility that combining the platforms to provide potentially complementary immune responses might provide better protection. There may also be benefit in combining ChAd63 MVA CS with other antigens, also delivered by the same viral vectors which shown to have protective efficacy [28].

Limitations of the study include the small numbers and fact that volunteers were malaria naïve. However both of these were requirements given that it was the first time that these products were administered to humans. There was no placebo group, however, these vectors, expressing a range of antigens, have been administered to over 460 individuals and have shown a consistent reactogenicity profile. The next stage in development is controlled human malaria challenge, where an unvaccinated control group is used.

### Conclusions

Given the excellent safety profile show here ChAd63 MVA CS should progress to Phase IIa efficacy study in adults with controlled human malaria infection. These vaccines could have a role in protection against malaria when used together, or combined with other vaccines.

## Supporting Information

S1 Figure **Gating strategy for analysis of CSP-specific T cell responses.** Representative flow cytometry plots are shown for the analysis of CSP-specific T cell responses from volunteers immunized with ChAd63-MVA CSP. (**A**) Initial gating used (from top left to bottom right) forward scatter area (FSC-A) versus forward scatter height (FSC-H) to remove doublet events and select singlet cells; then following this small lymphocytes were gated using FSC-A versus side scatter area (SSC-A); then live CD14^−^ CD20^−^ CD3^+^ cells were selected; then CD4 versus CD8 was used to select the total CD4^+^ CD8^−^ cell population and vice versa for the CD8^+^ CD4^−^ population. Cytokine (IFN-γ, IL-2 and TNFα) and CD107a gating using bivariate plots is shown for (**B**) CD4^+^ cells and (**C**) CD8^+^ cells. (**B**) Representative plots for un-stimulated (UNS), CS peptide stimulated (CS), SEB stimulated samples are shown. IFN-γ (top row), IL-2 (second row), TNFα (third row) and CD107a (bottom row) for the CD8^−^ CD4^+^ T cell population were analyzed using bivariate plots. Percentages refer to the % of CD8^−^ CD4^+^ cells that express the specific cytokine or marker. Background responses in UNS control cells were subtracted from the CS response respectively during the analysis. (**C**) Same analysis as in (**B**), except for the CD4^−^ CD8^+^ T cell population.
(TIF)

S1 Table **Assessment of severity of AEs.**
(TIF)

S2 Table **Assessment of severity of local AEs.**
(TIF)

S3 Table **Assessment of relationship of AE to vaccination.**
(TIF)

S4 Table **HLA typing of all volunteers enrolled in study.**
(TIF)

S5 Table **CSP overlapping peptides.** Peptides (NEO Peptide, Cambridge, MA, USA), 15 amino acids (aa) in length and overlapping by 10 aa spanning the entire CSP insert, were reconstituted in 100% DMSO at 50–200 mg/mL and combined into various pools for ELISPOT and flow cytometry assays. The composition of peptide pools containing to 2 to 15 peptides is shown here.
(TIF)

S6 Table **Demographic of study volunteers.**
(TIF)

S7 Table **Day 63 Multi-functional cells.** Percent of parent population (CD4+ or CD8+) producing 1 or more cytokine by group.
(TIF)

S1 Checklist **CONSORT Checklist.**
(DOC)

S1 File **Inclusion and exclusion criteria for volunteers in study.**
(PDF)

S2 File **Total IgG ELISA and IFA assays used.**
(PDF)

S1 Protocol **Clinical trial protocol.**
(PDF)
