# Supplementary figures and images for: A Phase Ia Study to Assess the Safety and Immunogenicity of New Malaria Vaccine Candidates ChAd63 CS Administered Alone and with MVA CS
Source: PLoS One. 2014 Dec 18;9(12):e115161. doi: 10.1371/journal.pone.0115161 (PMC4270740; doi:10.1371/journal.pone.0115161)

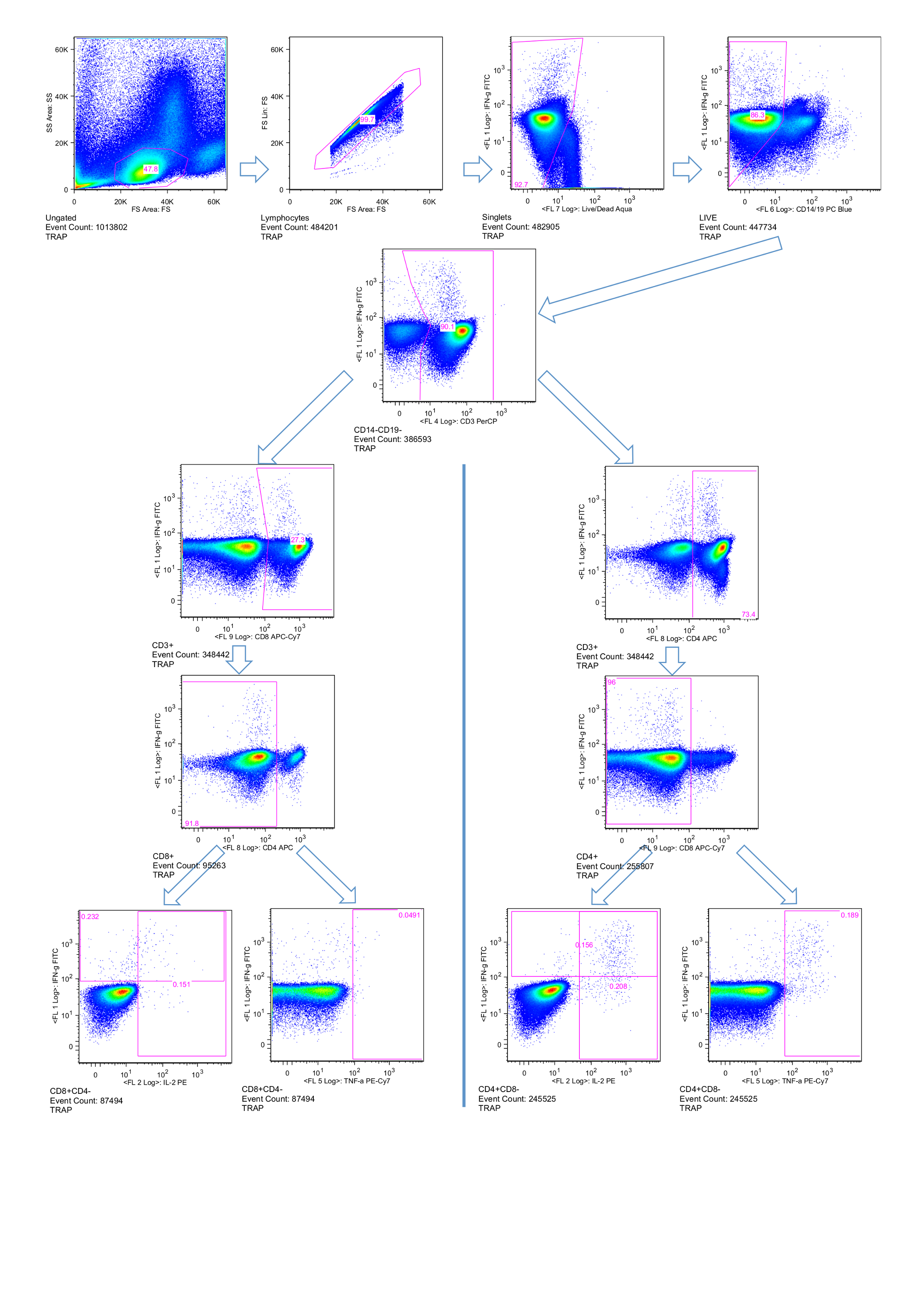

Supplement: S1 Figure — Gating strategy for analysis of CSP-specific T cell responses. Representative flow cytometry plots are shown for the analysis of CSP-specific T cell responses from volunteers immunized with ChAd63-MVA CSP. (A) Initial gating used (from top left to bottom right) forward scatter area (FSC-A) versus forward scatter height (FSC-H) to remove doublet events and select singlet cells; then following this small lymphocytes were gated using FSC-A versus side scatter area (SSC-A); then live CD14− CD20− CD3+ cells were selected; then CD4 versus CD8 was used to select the total CD4+ CD8− cell population and vice versa for the CD8+ CD4− population. Cytokine (IFN-γ, IL-2 and TNFα) and CD107a gating using bivariate plots is shown for (B) CD4+ cells and (C) CD8+ cells. (B) Representative plots for un-stimulated (UNS), CS peptide stimulated (CS), SEB stimulated samples are shown. IFN-γ (top row), IL-2 (second row), TNFα (third row) and CD107a (bottom row) for the CD8− CD4+ T cell population were analyzed using bivariate plots. Percentages refer to the % of CD8− CD4+ cells that express the specific cytokine or marker. Background responses in UNS control cells were subtracted from the CS response respectively during the analysis. (C) Same analysis as in (B), except for the CD4− CD8+ T cell population. (TIF) [file pone.0115161.s001.tif]
