# Supplementary material for: A Phase Ia Study to Assess the Safety and Immunogenicity of New Malaria Vaccine Candidates ChAd63 CS Administered Alone and with MVA CS
Source: PLoS One. 2014 Dec 18;9(12):e115161. doi: 10.1371/journal.pone.0115161 (PMC4270740; doi:10.1371/journal.pone.0115161)
Supplement: S1 File — Inclusion and exclusion criteria for volunteers in study. (PDF) [file pone.0115161.s010.pdf]

Supplementary Information S1:

Inclusion and Exclusion Criteria

Inclusion Criteria

The volunteer must satisfy all the following criteria to be eligible for the study:

- Healthy adults aged 18 to 50 years
- Able and willing (in the Investigator's opinion) to comply with all study requirements
- Willing to allow the investigators to discuss the volunteer's medical history with their General Practitioner
- Women only: Must practice continuous effective contraception for the duration of the study.
- Agreement to refrain from blood donation during the course of the study and for 6 months after the end of their involvement in the study.
- Written informed consent.

Exclusion Criteria

The volunteer may not enter the study if any of the following apply:

- History of clinical *P. falciparum* malaria
- Travel to a malaria endemic region during the study period or within the preceding six months with a significant risk of malaria exposure.
- Participation in another research study involving an investigational product in the 30 days preceding enrolment, or planned use during the study period.
- Prior receipt of an investigational malaria vaccine or any other investigational vaccine likely to impact on interpretation of the trial data.
- Administration of immunoglobulins and/or any blood products within the three months preceding the planned administration of the vaccine candidate.
- Any confirmed or suspected immunosuppressive or immunodeficient state, including HIV infection; asplenia; recurrent, severe infections and chronic (more than 14 days) immunosuppressant medication within the past 6 months (inhaled and topical steroids are allowed)
- Pregnancy, breast feeding or intention to become pregnant during the study
- History of allergic disease or reactions likely to be exacerbated by any component of the vaccine e.g. egg products, Kathon.
- History of clinically significant contact dermatitis.
- Any history of anaphylaxis post vaccination or any serious reaction following vaccination.
- History of cancer (except basal cell carcinoma of the skin and cervical carcinoma in situ).
- History of migraine headache.
- History of serious psychiatric condition that may affect participation in the study.

- Any other serious chronic illness requiring hospital specialist supervision. Use of regular medications such as antihypertensives would not necessarily result in exclusion.
- Suspected or known current alcohol abuse as defined by an alcohol intake of greater than 42 units every week or Carbohydrate Deficient Transferrin (CDT) >3%.
- Suspected or known injecting drug abuse in the 5 years preceding enrolment.
- Suspected or known use of opiates, cocaine, amphetamines, benzodiazepines or marijuana.
- Seropositive for hepatitis B surface antigen (HBsAg).
- Seropositive for hepatitis C virus (antibodies to HCV).
- Any clinically significant abnormal finding on biochemistry or haematology blood tests, urinalysis or clinical examination.
- Any other significant disease, disorder or finding which may significantly increase the risk to the volunteer because of participation in the study, affect the ability of the volunteer to participate in the study or impair interpretation of the study data.
  in a particularly dependent relationship with the investigator by way of occupation or otherwise, which in the investigators opinion places the volunteer in a vulnerable population.

Re-vaccination exclusion criteria

The following AEs associated with vaccine immunisation constitute absolute contraindications to further administration of an IMP to a volunteer. If any of these events occur during the study, the subject will be withdrawn from the trial and followed up by the clinical team or their GP until resolution or stabilisation of the event;

- Anaphylactic reaction following administration of vaccine
- Any serious reaction following vaccination
- Pregnancy

The following adverse events constitute contraindications to administration of vaccine at that point in time; if any one of these adverse events occurs at the time scheduled for vaccination, the subject may be vaccinated at a later date, or withdrawn, at the discretion of the investigator;

- Acute disease at the time of vaccination. (Acute disease is defined as the presence of a moderate or severe illness with or without fever.) All vaccines can be administered to persons with a minor illness such as diarrhoea, mild upper respiratory infection with or without low-grade febrile illness, *i.e.*, temperature of <37.5°C (99.5°F).
- Temperature of ≥37.5°C (99.5°F) at the time of vaccination.
