# Supplementary material for: A Phase Ia Study to Assess the Safety and Immunogenicity of New Malaria Vaccine Candidates ChAd63 CS Administered Alone and with MVA CS
Source: PLoS One. 2014 Dec 18;9(12):e115161. doi: 10.1371/journal.pone.0115161 (PMC4270740; doi:10.1371/journal.pone.0115161)
Supplement: S2 File — Total IgG ELISA and IFA assays used. (PDF) [file pone.0115161.s011.pdf]

Supplementary Information S2. Total IgG ELISA and IFA assays used.

Enzyme Linked Immunosorbent Assay (ELISA) methodology as follows; PfCSP ELISA: ELISA 96-well plates were coated with a synthetic peptide (Eurogentec) based on the repeat region of the PfCSP with the amino acid sequence CS(NANP)6C. The peptide was coated at a concentration of 0.2 µg/mL in a volume of 100 µL per well. Plates were placed inside a humidity chamber and incubated overnight (16 - 20 h) at 22C. Plates were washed four times with 1xPBS (pH 7.4) containing 0.5% Tween-20 and blocked with 0.5% casein blocking buffer (Sigma) for 1 h at 22 _C. Plates were washed four times and serially diluted samples were added and incubated at 22 _C for 2 h. After washing four times, peroxidase labelled goat anti-human IgG (KPL) was added at a dilution of 1:4,000 and incubated at 22C for 1 h. After washing four times, ABTS Peroxidase substrate (KPL) was added for development and incubated for 1h at 22C. The data were collected using Softmax Pro GXP, data were fit to a 4-parameter logistic curve, and the serum dilution at which the optical density was 1.0 (OD 1.0) calculated. To serve as a positive control, serum obtained from a volunteer participating in a Phase 1/2a challenge trial of R32NS181 formulated with alum was used. The individual had anti-PfCSP antibodies but was not protected (personal communication from WRAIR serology laboratory). Samples were considered positive if the difference between the post-immunization OD 1.0 and the pre-immunization OD 1.0 (net OD 1.0) was > 50 and the ratio of post- immunization OD 1.0 to pre-immunization OD 1.0 (ratio) was > 2. For example, if the OD 1.0 was 150 post-immunization and 50 pre-immunization, the net OD 1.0 would be 100, and the ratio of OD 1.0 post-immunization to OD 1.0 pre-immunization would be 3. This would be considered positive. For IFA, Analytic endpoint titer is the last dilution with measured lumens > LLD , Calculated endpoint titer is the extrapolated dilution where the lumens > LLD , BLD= Below the limit of detection in this assay (500) vs <500.

Preparation of Sporozoite Slides

NF54 mosquitoes were dissected. The thorax was cut at the scutum. The separated heads and the anterior portions of the scutum were kept while the remaining portions of the thorax and abdomen were discarded. The sporozoites were isolated using Ozaki tubes and then purified using a DEAE column. Sporozoites were counted using a hemocytometer. A sporozoite suspension of 5,000 sporozoites/well was added to each well. Slides were dried overnight. The slides were then wrapped in aluminum foil and placed in desiccator at -20ºC.

Immunofluorescence assay (IFA)

The negative control was obtained from a pool of normal rhesus sera and was diluted at 1:50 in PBS+1% BSA . The positive control was obtained from a subject immunized with NANP repeat peptide and also diluted at 1:50 in PBS +1% BSA . Slides were thawed for 20 minutes at room temperature and then blocked with16 µL of PBS +1% BSA. Then, 15 µL of each sample was added to each well and incubated for 1 hour in a humidity chamber. The slides were then washed 3 times for 2 minutes in 1XPBS. Next, 16 µL of the secondary antibody, goat anti-rhesus IgG (H+L) FITC (Cat# 6200-02, SouthernBiotech) diluted to 1:2000 with PBS+1% BSA was added to each well. The slide were incubated for 1 hour, then were washed 3 times for 2 minutes in 1XPBS. Finally, 1 µL of Vectashield with DAPI (Cat# H-1200, Vector laboratories) was added to each well and the slide was covered with a cover slip. The slide was covered with aluminum foil until read.

Image Analysis

Slides were read using an Olympus AX80 Provis fluorescent microscope with a 40x objective, exposure time of 1/3.5 seconds, an isotropic signal of 400 and at 1360x1024 resolution. The well was first scanned then a representative photograph was taken. Images were then analyzed using Image-Pro Plus, MediaCybernetics utilizing a software macro designed such that sporozoites were identified based on size and dimensions ignoring

luminous artifacts. Sporozoites were manually selected until all sporozoites were analyzed (a minimum of three). The luminosity for each parasite was automatically averaged and reported, maintaining a CV% less than 20%. Titer cut offs were determined based on the last dilution that gave a positive luminosity (Mean+2SD of the negative control).
