# Supplementary material for: A Phase Ia Study to Assess the Safety and Immunogenicity of New Malaria Vaccine Candidates ChAd63 CS Administered Alone and with MVA CS
Source: PLoS One. 2014 Dec 18;9(12):e115161. doi: 10.1371/journal.pone.0115161 (PMC4270740; doi:10.1371/journal.pone.0115161)
Supplement: S1 Protocol — Clinical trial protocol. (PDF) [file pone.0115161.s012.pdf]

Study Reference: VAC 038

PROTOCOL Version 4.0

30th March 2012

A Phase Ia Study to Assess the Safety and Immunogenicity of New Malaria Vaccine Candidates ChAd63 CS administered alone and with MVA CS

Chief Investigator: Professor A.V.S Hill

Sponsor: University of Oxford

Modification History

**Version**	**Date**	**Author(s)**
1.0	27th May 2011	Susanne Sheehy, Sam McConkey, Alison Lawrie, Sarah Gilbert, Adrian Hill.
2.0	21st July 2011	Eoghan de Barra, Susanne Sheehy, Sam McConkey, Adrian Hill.
3.0	11th Sept 2011	Eoghan de Barra, Alison Lawrie
4.0	30th March 2012	Eoghan de Barra, Alison Lawrie

Details of changes to Protocol from Version 1.0

Section	Details of change
4 Study Overview	Volunteer choice in group allocation removed. Investigator will allocate groups.
5.2	Clarification of role of DSMB
6.2 Informed consent	Requirement for GP communication and entry in TOPS database limited to UK volunteers only.
6.4 Withdrawal of volunteers	Added - Any volunteer who is withdraw or are withdrawn, post vaccination, will be invited to attend for all scheduled safety bloods and review as per protocol.
8.2 Secondary Evaluation Criteria	With reference to freezing and storage of samples for future investigations, this has been limited to UK volunteers only.
9.6 DSMB	Added -The DSMB will review the data before there is a dose escalation of ChAd63 CS from $5x10^9$ to $5x10^{10}$
10 Statistics	Added - Data analysis will consist primarily of descriptive summaries for treatment groups. For primary and secondary endpoints descriptive summaries and plots over the time course for both individual patient results and groups will be presented. Due the small number of volunteers in this study, all volunteers receiving the same dose of a given vaccine will be pooled for analysis. Where appropriate highly skewed data will be log-transformed and presented as geometric

	means with 95% confidence intervals.
6.3 Inclusion and Exclusion criteria	Added exclusion criteria; in a particularly dependent relationship with the investigator by way of occupation or otherwise, which in the investigators opinion places the volunteer in a vulnerable population. Removal of Oxford as a clinical site

A Phase Ia Study to Assess the Safety and Immunogenicity of New Malaria Vaccine Candidates ChAd63 CS administered alone and with MVA CS

Study Code: VAC 038

EudraCT Number: 2011-001875-38

**Chief Investigator**	Professor Adrian V.S. Hill<br>Centre for Clinical Vaccinology and Tropical Medicine<br>University of Oxford<br>Churchill Hospital, Old Road, Headington<br>Oxford, OX3 7LJ<br>Email: *adrian.hill@ndm.ox.ac.uk*
**Principal Investigator**	Professor Sam McConkey<br>Clinical Research Centre<br>Royal College of Surgeons in Ireland (RCSI)<br>Beaumont Hospital<br>Dublin 9<br>Ireland<br>Email: *smcconkey@rcsi.ie*
**Trial Sites**	Clinical Research Centre<br>Royal College of Surgeons in Ireland (RCSI)<br>Beaumont Hospital<br>Dublin 9<br>Ireland<br>PI at site: Professor Sam McConkey
**Sponsoring Institution**	University of Oxford<br>Clinical Trials and Research Governance<br>Manor House, The John Radcliffe Hospital,<br>Headington, Oxford, OX3 9DZ<br>Tel: 01865 743004<br>Fax: 01865 743002<br>Email: heather.house@admin.ox.ac.uk
**External Monitor**	Appledown Clinical Research Limited

Orchard's End,
Greenland's Lane,
Prestwood,
Gt. Missenden,
Bucks.
HP16 9QX
UK
Tel/Fax: +44 (0)1494 867676

Investigator Agreement

"I have read this protocol and agree to abide by all provisions set forth therein.

I agree to comply with the principles of the International Conference on Harmonisation Tripartite Guideline on Good Clinical Practice."

------------------------------	------------------------------	------------------------------
Chief Investigator	Investigator Signature	Date
Professor Adrian Hill		

Confidentiality Statement

This document contains confidential information that must not be disclosed to anyone other than the Sponsor, the Investigator Team, and members of the Independent Ethics Committee. This information cannot be used for any purpose other than the evaluation or conduct of the clinical investigation without the prior written consent of Professor Adrian Hill.

TABLE OF CONTENTS

1. SYNOPSIS....8

2. ABBREVIATIONS 10

3. BACKGROUND AND RATIONALE 11

4. Study overview....20

5. INVESTIGATIONAL PRODUCTS 23

6. RECRUITMENT AND WITHDRAWAL OF TRIAL VOLUNTEERS....25

7. TREATMENT OF TRIAL VOLUNTEERS 29

8. ASSESSMENT OF SCIENTIFIC OBJECTIVES 34

9. ASSESSMENT OF SAFETY 35

10. STATISTICS....42

11. QUALITY CONTROL AND QUALITY ASSURANCE PROCEDURES....43

12. ETHICS 45

13. DATA HANDLING AND RECORD KEEPING 46

14. FINANCING AND INSURANCE....47

15. references....48

1. SYNOPSIS

**Title**	**A Phase Ia Study to Assess the Safety and Immunogenicity of New Malaria Vaccine Candidates ChAd63 CS administered alone and with MVA CS**
**Trial Centres**	Clinical Research Centre, Royal College of Surgeons in Ireland (RCSI), Beaumont Hospital, Dublin 9, Ireland
**Trial Identifier**	VAC 038
**Clinical Phase**	Ia
**Design**	Open label observational study
**Population**	Healthy adults aged 18 – 50 years
**Sample Size**	Group 1<br>Subgroup A (1A): 4 volunteers;1 dose of ChAd63 CS 5 x 10^9 vp intramuscularly<br>Subgroup B (1B): 8 volunteers;1 dose of ChAd63 CS 5 x 10^9 vp intramuscularly and 1 dose MVA CS 2 x 10^8 pfu 8 weeks later intramuscularly<br><br>Group 2<br>Subgroup A (2A): 4 volunteers;1 dose of ChAd63 CS 5 x 10^{10} vp intramuscularly<br>Subgroup B (2B): 8 volunteers;1 dose of ChAd63 CS 5 x 10^{10} vp intramuscularly and 1 dose MVA CS 2 x 10^8 pfu 8 weeks later intramuscularly<br><br>Total: 24 volunteers
**Follow-up duration**	Minimum 6 months (This is an estimate and may vary in accordance with the specified time windows for each attendance)
**Planned Trial Period**	12 months
**Primary Objective**	To assess the safety of new candidate malaria vaccines ChAd63 CS administered alone and with MVA CS in a prime-boost regime to healthy volunteers.
**Secondary Objective**	To assess the humoral and cellular immune responses generated by ChAd63 CS when administered to healthy volunteers alone and with MVA CS.

**INVESTIGATIONAL PRODUCTS**	1. ChAd63 CS (Chimpanzee adenovirus 63 expressing circumsporozoite protein)<br>2. MVA CS (Modified vaccinia virus Ankara expressing circumsporozoite protein)
**Form**	Liquid
**Route of Administration**	Intramuscular (IM) needle injection into the deltoid region of the arm
**Dose per Administration**	- ChAd63 CS: 5×10^9 vp, 5×10^{10} vp<br>- MVA CS: 2×10^8 pfu

2. ABBREVIATIONS

**ChAd63**	Chimpanzee adenovirus 63
**AdHu**	Human adenovirus
**AdHu5**	Human adenovirus serotype 5
**AE**	Adverse event
**AMA1**	Apical membrane antigen 1
**CCVTM**	Centre for Clinical Vaccinology and Tropical Medicine
**CBF**	Clinical Bio manufacturing Facility
**CRF**	Case Report Form or Clinical Research Facility
**CS or CSP**	Circumsporozoite protein
**ELISPOT**	Enzyme-linked immunospot
**FBC**	Full blood count
**GCP**	Good Clinical Practice
**GIA**	Growth Inhibition Assay
**GMO**	Genetically modified organism
**HBsAg**	Hepatitis B Surface Antigen
**HCG**	Human Chorionic Gonadotrophin
**HCV**	Hepatitis C virus
**HIV**	Human immunodeficiency virus
**HLA**	Human leukocyte antigen
**IDT**	Impfstoffwerk Dessau-Tornau
**REC**	Independent Research Ethics Committee
**LSM**	Local safety monitor
**ME-TRAP**	Multiple epitopes and thrombospondin related adhesion protein
**MSP1**	Merozoite Surface Protein 1
**MVA**	Modified vaccinia virus Ankara
**pfu**	Plaque forming unit
**PMR**	Parasite Multiplication Rate
**REC**	Research Ethics Committee
**SAE**	Serious adverse event
**SOP**	Standard Operating Procedure
**SUSAR**	Suspected unexpected serious adverse reaction
**µg**	microgram
**vp**	viral particle

3. BACKGROUND AND RATIONALE

The need for a new vaccine against malaria

Although recent and encouraging evidence suggests that the epidemiology of *Plasmodium falciparum* malaria is changing across certain parts of Africa,[1] the worldwide burden of disease from malaria remains a major public health problem, with approximately 250 million cases and over 800,000 deaths worldwide in 2008, mostly in Africa.[2] The enormous economic and social consequences of malaria have been well documented.[3]

The development of resistance both in Anopheles mosquitoes to certain insecticides and of malaria parasites to chemotherapeutic agents has contributed to an increasing need for a new, effective intervention for the prevention or treatment of malaria.[4]

To provide a coordinated global approach to fighting malaria, the Roll Back Malaria (RBM) Partnership was launched in 1998 by the World Health Organization (WHO), the United Nations Children's Fund (UNICEF), the United Nations Development Programme (UNDP) and the World Bank. A major goal of the RBM Partnership is to support the development of a vaccine against malaria, felt to be a key future strategy for reducing mortality from malaria and moving towards eradication.[5]

Lifecycle of the malaria parasite

The malaria lifecycle is complex with stages in both human and mosquito hosts (Figure 1). The bite of infected female Anopheles mosquitoes transmits malaria sporozoites to the human host where they travel via the bloodstream to the liver and invade hepatocytes (*liver stage*). Here they mature into merozoites for 6 to 7 days after which the hepatocytes rupture releasing a large number of merozoites into the bloodstream. Merozoites then invade erythrocytes where they multiply and after 2 days cause the erythrocyte to rupture, releasing progeny merozoites that in turn invade new erythrocytes (*blood stage*). A small percentage of merozoites differentiate into gametocytes, which when ingested by a mosquito, unite with another gametocyte to create a zygote. The zygote matures and releases sporozoites which migrate to the mosquito's salivary glands and are injected into the human when the mosquito feeds. Infection by sporozoites and the liver-stage of malaria is asymptomatic. It is the blood stage of infection that is associated with symptoms and potentially severe or fatal complications.

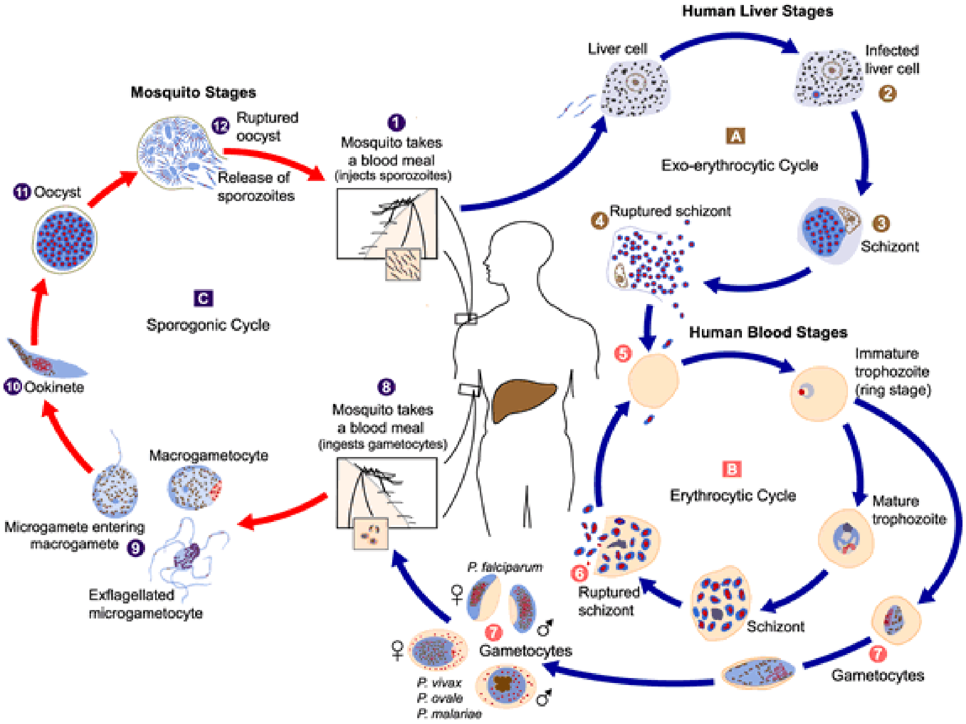

Figure 1 *Lifecycle of Malaria*

The Circumsporozoite Protein as a Vaccine Antigen

The structural and functional properties of CS were defined in the 1980's by the study of the mechanisms of the protective immunity induced by immunisation of rodents, monkeys and humans with sporozoites attenuated by irradiation.[6] Irradiated sporozoites invade hepatocytes but their further development is arrested. Protection appears to be dependent on the persistence of these arrested forms in the liver. Protection on sporozoite challenge in humans can be achieved following multiple, repeated bites from irradiated mosquitoes infected with P. falciparum, and this protection has been shown to be mediated by antibodies generated to CS.[7]

CS is expressed by sporozoites and liver schizonts and plays a key role in the attachment phase of sporozoite invasion into hepatocytes.[6] Anti-CS antibodies can target sporozoites, facilitating destruction of sporozoites prior to hepatocyte invasion. However, since sporozoites travel from the skin to liver within minutes, it may be difficult for a vaccine to achieve complete protection against *P. falciparum* sporozoites based solely on antibodies. The liver stage of infection provides a longer window of opportunity for cell mediated immunity to recognise and destroy infected hepatocytes.

In order to induce T cell recognition, liver stage antigens need to be processed and presented on the surface of infected hepatocytes. A significant obstacle is that the liver stages reside inside a parasitophorous vacuole surrounded by a membrane that is only permeable to small molecules. To date, only CS has been found in the cytoplasm of infected hepatocytes, confirming the importance of CS as a target antigen in liver stage immunity.[6] Importantly, sporozoites also secrete CS while they glide through or traverse the endothelium. Thus, CD4+ T cells may recognize processed CS on the plasma membrane of non-parenchymal liver cells, such as Kupffer cells or dendritic cells that express constitutively class II MHC. If T cell recognition is followed by release of cytokines such as interferon-γ in the proximity of the infected hepatocyte, then the liver stage development will be inhibited.

Progress towards a pre-erythrocytic malaria vaccine

The candidate pre-erythrocytic malaria vaccine RTS,S is the most advanced and efficacious malaria vaccine in development.[6,8] It is formed from the fusion of CS to the surface antigen of hepatitis B virus to form virus like particles. This construct, administered with proprietary adjuvants is currently in phase III studies in multiple sites in African infants, where it has been shown to be safe, immunogenic and efficacious, inducing approximately 45% clinical efficacy which persists up to 15 months.[9] Whilst these clinical results are the most effective for any malaria vaccine product to date, there remains considerable capacity and need to improve on this limited clinical efficacy, either through modifications to the RTS,S vaccine or by developing vaccine strategies that combine numerous antigens or vaccine strategies.

Analysis of the immunological correlates of immunity induced by the RTS,S/AS02 vaccine in both phase IIa sporozoite challenge studies[10,11] and a more recent trial in Mozambique[12] provide evidence that very high levels of antibodies to CS correlate with protection in humans. However, this correlation is relatively weak and there may be a component of T cell mediated protection induced by the vaccine, even though the magnitude of the T cell response measured after vaccination is modest (approximately 150 SFU / million PMBCs on ELIspot).[13]

Increasing data from animal models, fieldwork and inoculation of volunteers with irradiated sporozoites support an important role for T cells, in particular $CD8^+$ cells, in mediating pre-erythrocytic immunity, even in the absence of antibodies.[14] Whilst pre-clinical studies demonstrate a clear correlation between $CD8^+$ T cells and protection,[15-19] clinical vaccine studies have been hampered by the limited ability of existing vaccine strategies, namely adjuvanted protein constructs, to induce high enough numbers of antigen specific $CD8^+$ T cells to confer protection.

Adrian Hill's group at the University of Oxford have been working for over 10 years to develop a pre-erythrocytic P. falciparum malaria vaccine using the sporozoite and liver stage antigen ME-TRAP. This antigen contains a fusion protein of multiple epitopes (ME; a string of 20 epitopes, mainly CD8+ T cell epitopes from pre-erythrocytic antigens) and the *P. falciparum* pre-erythrocytic antigen; thrombospondin-related adhesion protein (TRAP).[20]

Multiple vectors for this antigen have been clinically tested including DNA, fowl pox (FP) and modified vaccinia virus Ankara (MVA), however T cell immunogenicity and clinical efficacy has been limited (Table 1). Most recently. heterologous prime boost with Chimpanzee adenovirus 63 (ChAd63) and MVA ME-TRAP has been shown to be the most immunogenic regimen to date, inducing more than 2400 IFNγ producing T cells post boost (Figure 3, O'Hara et al submitted). Sporozoite challenge of malaria naïve individuals vaccinated with ChAd63-MVA ME-TRAP demonstrated significant clinical efficacy of this vaccine strategy, with 3/14 individuals demonstrating sterile protection (21%) and 5/14 demonstrating partial protection (36%) (Figure 2A, Ewer et al submitted). Of note, on re-challenge 8 months later, all 3 sterilely protected volunteers demonstrated evidence of persisting protection, with 1 volunteer demonstrating sterile protection and the other two partial protection. In this study, protection was shown to correlate strongly with mono-functional $CD8^+$ T cells (Figure 2B).

Vaccine encoding ME-TRAP	T cell response mean cells/million PBMCS*	Protective Efficacy on Sporozoite Challenge
DNA x 3[21]	48	Nil
Fowl-pox x 2	50	Nil
MVA x 3[21]	41	Nil
DNA & MVA[21,22]	430	23%
Fowl-pox & MVA[23]	475	25%
ChAd63-MVA*	2400	58%

Table 1: *Clinical trials of ME-TRAP encoding vaccines by University of Oxford, summarizing maximum T cell response as measured by IFNγ producing ELIspot at peak time point post final boost, and clinical efficacy as measured on sporozoite challenge. *Ewer et al Submitted.*

A B

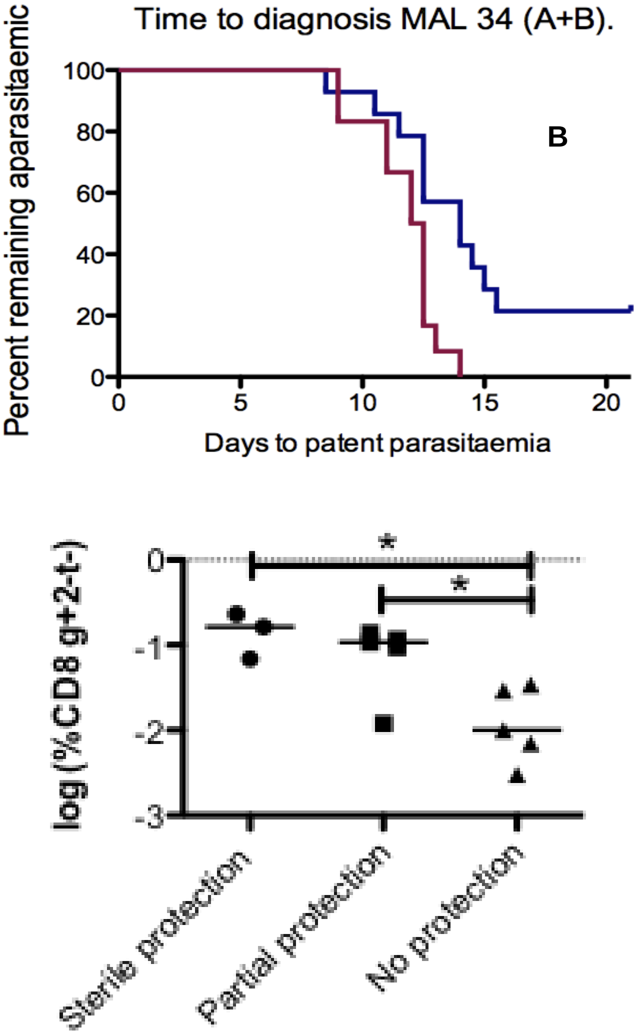

Figure 2: *Data from 14 healthy malaria naïve adult volunteers vaccinated with 5 x 10^{10} vp ChAd63 ME-TRAP intramuscularly, followed 8 weeks later by 2 x 10^{8} pfu MVA ME-TRAP intradermally.* ***Figure 2A:*** *Clinical Efficacy on heterologous sporozoite challenge with 3D7 P. falciparum conducted in 2 phases. The 12 control volunteers (red line) were all diagnosed with malaria. 57% of vaccines (blue line) demonstrated clinical efficacy; 3/14 volunteers demonstrated sterile protection, 5/14 volunteers demonstrated partial protection (a delay in time to diagnosis).* ***Figure 2B:*** *Vaccinees with both sterile and partial protection had higher significantly higher CD8+ levels than non-protected vaccinees.*

Given the proven clinical efficacy with a vaccine encoding CS, evidence of the importance of CD8+ responses in liver stage immunity and the ability of ChAd63-MVA to induce exceptionally potent $CD8^+$ T cells in addition to good humoral responses, the next logical step is to develop and test ChAd63-MVA expressing CS. This vaccine regimen could then be combined with ChAd63-MVA expressing ME-TRAP in order to increase clinical efficacy. Alternatively, ChAd63-MVA CS could be combined with the current leading vaccine RTS,S.

To date there have been a number of attempts to combine RTS,S with viral vectors vaccines; Firstly, a phase I/IIa trial of heterologous prime-boost immunization of RTS,S/AS02 and MVA encoding the entire CS gene construct (CSO) was undertaken in Oxford.[24] In this trial MVA vectored CS, known as MVA CSO was only modestly immunogenic and did not appear to enhance the efficacy of the RTS,S vaccine, although statistical power to assess this was limited. Particularly disappointing was the inability of the prime-boost approach to enhance the T cell immunogenicity to levels greater than RTS,S/AS02 alone. A further phase I/IIa trial of MVA and FP vectors expressing CSO demonstrated only modest T cell immunogenicity and no efficacy on sporozoite challenge.[25]

An improved insert design for CSP

The poor immunogenicity of the standard full length CSP insert used in previous vectors in clinical trials (CSO),[22,25-27] suggest that there may be an important difference in the intrinsic immunogenicity of CSO compared to the ME-TRAP insert. Using information from multiple sources,[28-30] we have designed a novel CS antigen, to be used in this study, which omits the extreme C-terminus of the protein that encodes the GPI-anchor sequence and the N-terminal third of the protein N-terminal to the central B cell repeat (see IMP-D for more details). Coincidentally, this creates a sequence encoding amino acids very similar to those of the RTS,S protein (the 'repeat' region of CSP consists of multiple repeats of NANP and NVDP. RTS,S contains 16 copies of NANP and none of NVDP. CS contains 13 copies of NANP and 3 of NVDP. The 'T cell epitope' region is 100 % identical at the amino acid level in the two sequences). We are confident that use of this novel antigen in the vectors ChAd63 & MVA will be more successful that the CSO antigen used in DNA, MVA and FP9 vectors to date.[22,25-27]

Clinical Trials of CSP Vaccines

Other than RTS,S the only other vaccine candidates targeting the CS protein currently in clinical development are human adenovirus 35 (Ad35) expressing CS from Crucell and human adenovirus 5 (Ad5) from the US Military Malaria Vaccine Program. This Crucell vaccine developemnt programme is sponsored by the US National Institute of Allergy & Infectious Diseases (NIAID) who are currently conducting Phase Ia & Phase 1b studies of Ad35 CSP administered in homologous prime boost regimens in adults. Data on these trials have yet to be published. (ClinicalTrials.gov identifiers: NCT01018459 & NCT00371189). The US Navy has undertaken unpublished clinical studies of Ad5 CS used alone and in a DNA prime-Ad5 prime-boost regimen. Vaccinees administered Ad5 CS alone reportedly failed to show efficacy against sporozoite challenge. However, in a prime boost regime where CS and AMA1 encoding vectors were mixed prior to administration a regime of DNA vector priming and Ad5 boosting led to 4 out of 15 vaccinees showing sterile protection against sporozoite challenge (T Richie personal communication). This result support further assessment of the utility of CS-based vectors particularly in heterologous prime-boost regimes.

Adenoviruses as Vectors

Adenoviruses are attractive viral vectors as they possess a genetically stable virion (so that inserts of foreign genes are not deleted), they can infect large numbers of cells and the transferred information remains epichromosomal, thus avoiding any potential for insertional mutagenesis. Replication defective adenovirus can be engineered by deletion of genes from the E1 locus, which is required for viral replication, and these viruses can be propagated easily with good yields in cell lines expressing E1 from AdHu5 such as human embryonic kidney cells 293 (HEK 293).[31] Previous mass vaccination campaigns using orally administered live human adenovirus serotype 4 and 7 in large numbers of US military personnel have shown good safety and efficacy data.[32]

Human adenoviruses have been used as vaccine vectors for a number of conditions, however a limiting factor to widespread use has been the level of anti-vector immunity present in humans where adenovirus is a ubiquitous infection. Estimates suggest that depending on the geographical region between 45–80% of adults carry AdHu5-neutralising antibodies.[33] Immunisation with AdHu vectors in animal models in the presence of pre-exposure to human adenoviruses attenuates responses to the vaccine probably due to the removal of virus particles by pre-existing antibodies.[34-36] Phase I trials of a multiclade HIV-1 vaccine delivered by a replication defective AdHu5 had to exclude volunteers with pre-existing antibodies to AdHu5 at titres greater than 1:12.[37] In recent Phase I placebo controlled human trials of a modified AdHu5 HIV vaccine there were no safety concerns amongst vaccinated volunteers with pre-existing high titre anti-AdHu5 antibodies, indeed less reactogenicity was seen amongst those with high-titre antibodies.[38] Using AdHu5 in a prime boost strategy for HIV-1 gag homologous boosting did not improve the peak post prime levels of gag specific lymphocytes, probably due to anti-vector immunity.[39]

The prevalence of immunity to human adenovirus prompted the consideration of simian adenoviruses as vectors. They exhibit hexon structures homologous to that of human adenoviruses.[40] Indeed, the chimpanzee adenovirus ChAd63's hexons are most similar in sequence to the hexons of AdHu4 previously used by the US military in mass vaccination campaigns where over 2 million adults received tablets of serially passaged adenovirus with good safety and efficacy data (Personal Communication Col. John D. Grabenstein).[41] In chimpanzee adenoviruses the E1 locus can be deleted to render viruses replication deficient and allow transcomplementation on an E1 AdHu5 complementing cell line.[42] An additional attractive observation is that the lack of sequence homology between AdHu5 and simian adenoviruses at the E1 flanking sequence prevents homologous recombination and production of replication competent virus.[43]

Simian adenoviruses are not known to cause pathology or illness in humans and the prevalence of antibodies to chimpanzee origin adenoviruses is less than 5% in humans residing in the US.[44] In Equatorial Africa (the natural habitat for chimpanzees), prevalence is higher but still below that to anti AdHu5 immunity. In a recent study in Kenya, 23% of children aged 1-6 years had neutralising antibodies at a titre greater than 1:200 to AdHu5, whilst only 4% had high-titre neutralising antibodies to ChAd63. Immunity to both vectors was age-dependent.[45] Early murine work using chimpanzee adenovirus 68 (AdCh68, also called C9) expressing *gag* of HIV-1 showed that in comparison to AdHu5 and poxvirus, AdCh68 was as effective at generating a transgene product specific CD8+ T cell response with approximately 20% of all splenic CD8+ being gag specific.[46] In the same study, pre-exposure to AdHu5 abolished any protection offered by immunisation with AdHu5 but only slightly reduced that elicited by AdCh68, suggesting pre-exposure to human adenoviruses should not reduce the potency of the immune response generated to simian vectored vaccines.

There is no available or validated in vitro cell co-culture method to examine co-infection with human and simian adenovirus vectors as the latter are non-replicating. Due to a lack of any sequence homology between the replication-deficient ChAd63 and MVA vectors, complementation of MVA by ChAd63 does not occur. Pre-clinical bioavailability studies have demonstrated no persistence of the ChAd63 vector 24 hours post intramuscular administration. Therefore, residual priming ChAd63 vector is very unlikely to be present at the time of administration of a MVA boost, 8 weeks later.

Chimpanzee Adenovirus 63 (see also ChAd63 CS Investigator Brochure)

ChAd63 expressing varying antigens has been administered to over 250 individuals including 24 Gambian children (Table 2) and has demonstrated an excellent safety profile, with doses of up to 2 x 10^{11} vp ChAd63 ME-TRAP found to be safe in UK adults. The vector has been shown to be consistently immunogenic, inducing extremely potent T cell responses (Figure 3) *and* good antibody responses, especially when combined with MVA. Multiple studies have shown 5 x 10^{10} vp ChAd63 to be the optimal dose, associated with a consistently excellent reactogenicity profile and potent T cell immunogenicity (see investigator brochure).

	ChAd63 ME-TRAP	ChAd63 MSP1	ChAd63 AMA1
Adults in UK	108	45	34
Adults in Africa	46	0	0
Children in Africa (2-6 years)	24	0	0
Total No. of Individuals Vaccinated	178	45	34

Preferred Dose in Adults	5 x 10^{10} vp	5 x 10^{10} vp	5 x 10^{10} vp
Preferred Dose in Children	5 x 10^{10} vp	Not Known	Not Known

Table 2: *Numbers of individuals vaccinated to date with ChAd63 vectored vaccines. Total: 257 individuals. ME-TRAP = Multiple epitopes + thrombospondin-related adhesion protein, MSP1 = Merozoite surface antigen 1, AMA1 = Apical Membrane Antigen 1.*

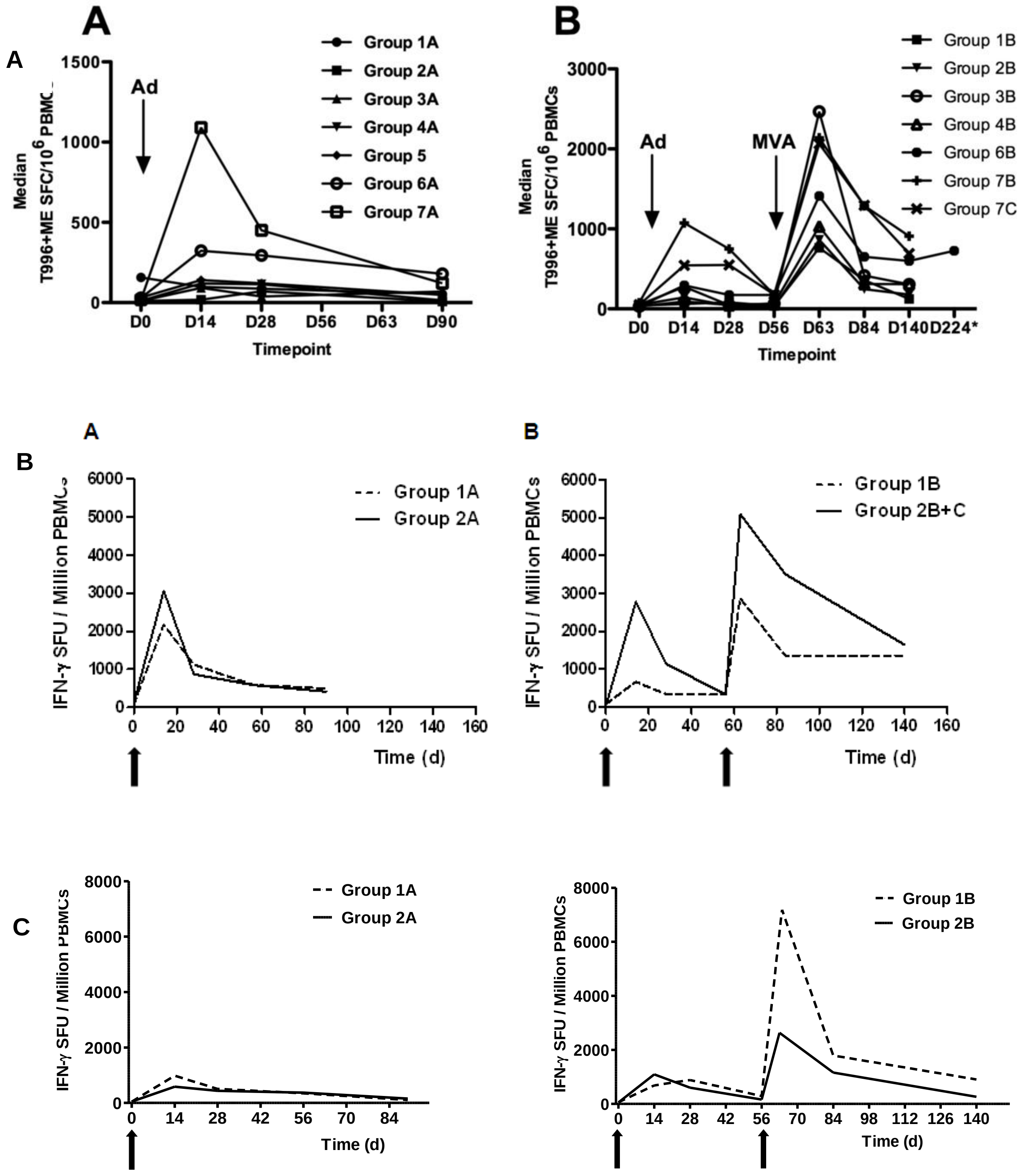

Figure 3: *T cell immunogenicity as measured by no. of antigen specific T cells measured by interferon γ ELIspot. Group A = ChAd63 priming vaccination only. Group B = ChAd63 prime & MVA boost. Increasing group number is associated with increasing dose of ChAd63. Priming vaccination takes place on Day 0, boost vaccinations on Day 56.* ***Figure 3A:*** *ChAd63-MVA ME-TRAP. Dose escalation of ChAd63 ME-TRAP, Dose of MVA ME-TRAP constant; 2 x 10^8 pfu.* ***Figure3B:*** *ChAd63-MVA MSP1. Dose of ChAd63 MSP1; 5 x 10^9 vp & 5 x*

10^{10} vp. Dose of MVA MSP1; 5 x 10^8 pfu. ***Figure 3C:*** *ChAd63-MVA AMA1. Dose of ChAd63 AMA1; 5 x 10^9 vp & 5 x 10^{10} vp. Dose of MVA AMA1;variable.*

Concerns exist that pre-existing antibodies to ChAd63 could limit wide spread use of the vector. However, data from the Phase IIb efficacy study of ChAd63-MVA ME-TRAP showed no correlation between neutralising antibodies to ChAd63 in volunteers prior to vaccination and their subsequent T cell count post MVA boost, suggesting that even if neutralising antibodies exist they may not limit immunogenicity (Figure 4, Ewer et al, submitted). There is no evidence that pre-existing neutralising antibodies to ChAd63 increase reactogenicity (O'Hara et al. submitted).

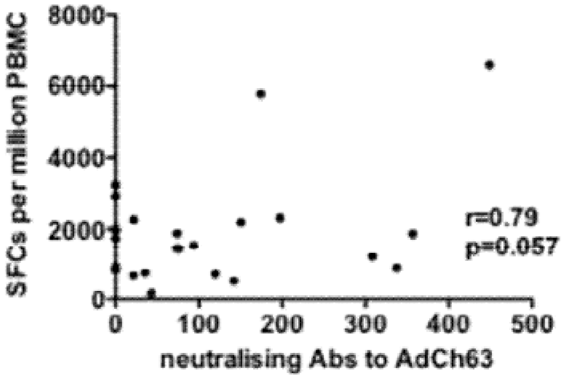

Figure 4: *Correlation between neutralising antibodies to ChAd63 measured prior to vaccination and total number of T cells measured by interferon γ ELIspot post ChAd63-MVA ME-TRAP in 21 malaria naïve healthy adults living in the UK.*

MVA as a Vector (See also MVA CS Investigator Brochure)

MVA is an attractive candidate orthopox vaccine vector for safety and immunogenicity reasons. The successful worldwide eradication of smallpox using vaccination with vaccinia virus highlighted vaccinia as a candidate carrier. Although millions of humans have been vaccinated with conventional replication-competent vaccinia virus, its small but definite risk to both researchers and future patients led to the development of several attenuated strains of vaccinia during smallpox eradication and more recently. In particular the host-range restricted MVA proved to be extremely attenuated compared to other vaccinia viruses.

MVA was originally derived from the vaccinia strain Ankara by over 500 serial passages in primary chicken embryo fibroblasts (CEF cells). MVA has six major genomic deletions compared to the parental Ankara genome and is severely compromised in its ability to replicate in mammalian cells. No replication has been documented in non-transformed mammalian cells. The viral genome has been proven to be stable through a large series of passages in chicken embryo fibroblasts.[47] MVA also showed no cytopathic effect or plaque formation in cells of human origin. In irradiated mice, MVA did not elicit any morbidity or lethality even when administered at high doses intra-cerebrally, indicating its safety even in immuno-compromised organisms.[47]

Apart from studies in mice, rabbits and elephants[48], MVA has been shown to be safe in humans.[49] From 1972 until 1980 (the end of compulsory smallpox vaccination) MVA was licensed in Germany[48] and was included in the official immunisation schedule.[50] In a large field study carried out in Germany in the late seventies, over 120,000 previously unvaccinated individuals were vaccinated with MVA (0.2 mL) administered either intra-dermally or subcutaneously. The study population included high-risk groups such as people suffering from allergies, elderly people and alcoholics. Given intradermally, a red nodule of up to 4 mm in diameter was observed at the injection site at day 4 or 5. Only a small proportion showed any systemic side effects such as fever > 38.5C[47]. MVA proved to be non-contagious and avirulent. Viral replication is blocked late during infection of cells but

importantly viral and recombinant protein synthesis is unimpaired even during this abortive infection. Replication-deficient recombinant MVA has been viewed as an exceptionally safe viral vector. When tested in animal model studies, recombinant MVAs have been shown to be avirulent, yet protectively immunogenic as vaccines against viral diseases and cancer[47]. Recent studies in macaques severely immuno-suppressed by SIV infection have further supported the view that MVA should be safe in immuno-compromised humans.

MVA is currently in development as a vector for multiple diseases including HIV,[53,54] Tuberculosis,[55] Hepatitis C (Barnes et al submitted), influenza[56] and melanoma.[57] MVA vectored vaccines are particularly suited to boosting immune responses to an antigen following a priming vaccination with another viral vector.[49] In Professor Hill's group, MVA encoding the malaria antigens has been administered to over 940 individuals, including children and infants in sub Saharan Africa (Table 3). The optimal dose of MVA has been shown consistently to be 1-2 x 10^8 pfu. Clinical studies have shown intramuscular administration to be associated with fewer and short lived local AEs and no reduction in immunogenicity (O'Hara et al submitted).

	**MVA ME-TRAP**	**MVA CSO**	**MVA polyprotein**	**MVA MSP1**	**MVA AMA1**
Adults in UK	165	78	28	32	26
Adults in Africa	308	71	0	0	0
Children and Infants in Africa	238	0	0	0	0
Total	711	149	28	32	26
Total post ChAd63 prime	134	0	0	32	26
Preferred Dose in Adults	2 x 10^8 pfu	1-2 x 10^8 pfu	1-2 x 10^8 pfu	2 x 10^8 pfu	1.25 x 10^8 pfu
Preferred dose in Children	2 x 10^8 pfu	1-2 x 10^8 pfu	Not known	Not known	Not Known

Table 3: *Total numbers of individuals vaccinated to date with MVA vectored malaria vaccines developed by the Hill group, University of Oxford. Total: 946 individuals. ME-TRAP = Multiple epitopes + thrombospondin-related adhesion protein, MSP1 = Merozoite surface antigen 1, AMA1 = Apical Membrane Antigen 1.*

4. STUDY OVERVIEW

This is an open label phase Ia clinical trial to assess the safety and immunogenicity of different doses of ChAd63 CS administered alone and with MVA CS in a heterologous prime-boost regimen. All volunteers recruited will be healthy adults aged between 18 and 50. Volunteers will be allocated to the groups by the investigators. Safety data will be collected for each of the regimens (Table 4). The immune responses generated by each of these regimens will be assessed.

4.1 Objectives

Primary Objective

- To assess the safety in healthy volunteers of two different doses of ChAd63 CS administered alone and with MVA CS in a heterologous prime boost regimen.

Secondary Objective

- To assess the immunogenicity in healthy volunteers of two different doses of ChAd63 CS administered alone and with MVA CS in a heterologous prime boost regimen.

4.2 Study Groups

Group Number	No. of volunteer s	ChAd63 CS Day 0	MVA CS Day 56
1A	4	5×10^9 vp	-
1B	8	5×10^9 vp	2×10^8 pfu
2A	4	5×10^{10} vp	-
2B	8	5×10^{10} vp	2×10^8 pfu

Table 4: *Overview of trial groups: All vaccinations are intramuscular.*

4.3 Rationale for Trial Design

Administration Schedules

Heterologous prime boost with ChAd63-MVA is, to our knowledge, one of the most potent T cell inducing subunit vaccine regimens which can importantly also induce antibodies. Previous clinical trials using this regimen expressing ME-TRAP, AMA1 & MSP1, have shown that administering ChAd63 as a prime followed 8 weeks later by MVA as a boost is a very immunogenic schedule (O'Hara et al submitted, Sheehy et al submitted). For this reason, and to provide comparability with previous ChAd63-MVA trials we propose to use a similar administration schedule.

Route & Dose

Our choice of the dose and route of vaccines in this study is based on experience using the same vectors in previous Phase I and Phase II clinical trials in the UK and Africa (see investigator brochures) using both intradermal and intramuscular routes.

We have chosen here the intramuscular route of administration for all vaccines given the proven favourable safety and immunogenicity profile of this route of administration with these vectors and because of future practical considerations regarding administration in the field.

ChAd63 has been safety administered to more than 250 healthy individuals and the vector has been shown repeatedly to be safe at the planned dose of 5 x 10^{10} vp (see investigator brochure).

MVA has been administered to more than 120 healthy UK adults following priming with ChAd63 expressing the same antigen, at various doses. Repeatedly, a dose of 1-2 x 10^{8} pfu MVA has been found to be a suitable dose to balance immunogenic and reactogenicity (see investigator brochure).

4.4 Duration of Study

Groups 1A & 2A

The duration of involvement in the study from enrolment will be approximately 6 months.

Groups 1B & 2B

The duration of involvement in the study from enrolment will be approximately 6 months.

4.5 Definition of the Start and End of the Trial

The start of the trial is defined as the date of the first vaccination of the first volunteer. The end of the trial is the date of the last visit of the last volunteer.

4.6 Potential Risks & Benefits for Volunteers

POTENTIAL RISKS

Phlebotomy: The maximum volume of blood drawn over the study period (572 mls over approximately 5 months) should not compromise these otherwise healthy volunteers. There may be minor bruising, local tenderness or pre-syncopal symptoms associated with venepunture, which will not be documented as AEs if they occur.

Vaccination: Potential expected risks from vaccination, which include local and systemic reactions are specific to each IMP and are described below. It is important to note that ChAd63 CS & MVA CS have not previously been administered to humans. Therefore, although the AE profile can be estimated from previous use of these vectors, the reactogenicity may vary from that seen previously with ChAd63 and MVA encoding different antigens. For this reason, vaccinees will be enrolled in a staggered format (Section 5.2) to allow early identification of any concerning reactogenicity before the majority of individuals have been vaccinated.

As with any vaccine, Guillain-Barré syndrome or immune-mediated reactions that can lead to organ damage including serious allergic reactions may occur but this should be extremely rare. Serious allergic reactions including anaphylaxis could also occur and for this reason volunteers will be vaccinated in a clinical area where Advanced Life Support trained physicians, equipment and drugs are immediately available for the management of any serious adverse reactions.

1. ChAd63 CS

Although ChAd63 CS has not previously been administered to humans, the safety data available from the more than 250 individuals who have previously received ChAd63 vectored vaccines can be used to predict the adverse event profile expected following vaccination with ChAd63 CS in this study; Local adverse events such as pain would be expected to occur frequently. Less frequent adverse events are likely to include erythema, swelling, itching and warmth. Local AEs are likely to be mild in nature and should resolve rapidly, although there is the possibility of moderate or severe arm pain in some cases.

Common systemic adverse events post viral vectored vaccines include headache, feverishness, myalgia, arthralgia, fatigue and malaise. Generally volunteers report a transient flu like illness within 24 hours of vaccination with ChAd63 which resolves completely within 48hrs. The majority of systemic AEs are likely to be mild but there is a possibility of moderate or severe headache or malaise. Given existing data for ChAd63 vectored vaccines, it is anticipated that the majority of systemic adverse events post ChAd63 CS will be mild in intensity.

During the manufacturing process of ChAd63 CS, a biocide named Kathon is used. Kathon is added to body washes, conditioners, liquid soaps, shampoos and wipes as a preservative. The maximum dose is 0.1% for 'rinse off' products and for 'leave on' products it is 0.05%. It has been approved by regulatory authorities throughout the world as a preservative in these products. As a skin sensitiser it is known to cause contact dermatitis. An internal study was set up to look at the levels of Kathon that were removed during the final purification step of buffer exchange. This study utilized high performance liquid chromatography and showed that trace amounts of Kathon may be left on the column after carrying out the rinse and sanitisation steps. However, the study confirmed greater than 99.9975% removal of Kathon to approximately 30 fold less than the limits for 'leave on' products containing Kathon. We will exclude anyone from the study with a history of clinically significant contact dermatitis or sensitivity to Kathon.

2. MVA CS

Although MVA CS has not previously been administered to humans, the safety data available from the more than 160 UK adults who have been boosted with MVA expressing malaria antigens following ChAd63 prime, (particularly those for intramuscular administration and lower doses of MVA) can be used to predict the adverse event profile expected post vaccination with MVA CS in this trial. At the planned dose (2×10^8 pfu), it is expected that majority of injection site reactions will be of mild severity. Injection site pain would be expected to occur frequently. Less frequent adverse events are likely to include erythema, swelling, itching and warmth. Local AEs are likely to be mild in nature and should resolve rapidly, although there is the possibility of moderate arm pain in some cases.

Common systemic adverse events post MVA vectored vaccines include headache, feverishness, myalgia, arthralgia, fatigue, and malaise. Generally volunteers report a transient flu like illness within 24 hours of vaccination with MVA which resolves completely. Given existing data for MVA vectored vaccines in Oxford, it is anticipated that the majority of systemic adverse events post 2×10^8 pfu MVA CS will be mild or moderate in intensity.

POTENTIAL BENEFITS

Volunteers will not benefit directly from participation in this study. However, it is hoped that the information gained from this study will contribute to the development of a safe and effective malaria vaccine regimen. The only benefits for participants would be information about their general health status.

5. INVESTIGATIONAL PRODUCTS

ChAd63 CS

ChAd63 CS was manufactured under Good Manufacturing Practice conditions by the Clinical Biomanufacturing Facility (CBF), Churchill Hospital, Oxford. ChAd63 CS is supplied as a liquid in sterile aliquots in 2.0 mL clear glass vials. Further details relating to batch release and manufacturing can be found in the ChAd63 CS IMP-D. The concentration of ChAd63 CS is 1.4 x 10^{11} VP/ml and therefore a dilution will be performed to achieve the lower dose of 5 x 10^{9}VP. According to SOP MC012.

MVA CS

MVA CS was manufactured under Good Manufacturing Practice conditions by Impfstoffwerk Dessau-Tornau (IDT) Germany. MVA CS is supplied as a liquid formulation in Tris buffer. The virus suspension is supplied as sterile aliquots in 2.0 mL clear glass injection vials. Final batch certification and associated labelling takes place at the Clinical Biomanufacturing Facility (CBF), Churchill Hospital, Oxford. A dose of 2 x 10^{8} will be administered as a volume of 0.34 ml from 2 vials of MVA CS at concentration of 5.9 x 10^{8} pfu/ml.

5.1 Storage of Vaccines

Vials of ChAd63 CS and MVA CS will be stored between –70^{0}C and –90^{0}C. All movements of the study vaccines will be documented. Vaccine accountability, storage, shipment and handling will be in accordance with local SOPs and other relevant local forms.

5.2 Administration of Vaccines

The vaccines will all be administered intramuscularly. The vaccinating investigator will wear gloves and eye protection. During administration of the vaccines, Advanced Life Support drugs and resuscitation equipment will be immediately available for the management of anaphylaxis. Vaccination will be performed and the IMP handled according to the relevant local SOPs. On vaccination day, vaccines will be allowed to thaw to room temperature and administered within 1 hour. Depending on dose, one or more vials of vaccine may be used.

Administration of ChAd63 CS

The first volunteer in group 1 (from group 1A or group 1B) will be vaccinated alone with 5 x 10^{9} vp ChAd63 CS. In the absence of any safety concerns in this first volunteer at least 72 hrs post vaccination, two further volunteers in group 1 (from group 1A or group 1B) will be vaccinated with 5 x 10^{9} vp ChAd63 CS. In the absence of any safety concerns in these two vaccines at least 72 hrs post vaccination, the remaining volunteers in group 1 will be vaccinated with 5 x 10^{9} vp ChAd63 CS.

There will be a minimum interval of two weeks between the administration of 5 x 10^{9} vp ChAd63 CS to the last volunteer in group 1 and administration of 5 x 10^{10} vp ChAd63 CS to the first volunteer in group 2. The DSMB will the review the safety data from the low dose volunteers prior to dose escalation to the higher dose vaccine.

The first volunteer in group 2 (from group 2A or 2B) will be vaccinated alone with 5 x 10^{10} vp ChAd63 CS. In the absence of any safety concerns in this first volunteer at least 72 hours post vaccination, two further volunteers in group 2 (group 2A or 2B) will be vaccinated with 5 x 10^{10} vp ChAd63 CS. In the absence of any safety concerns in these two vaccinees in group 2 at least 72 hours after vaccination, the remaining volunteers in group 2 will be vaccinated with 5 x 10^{10} vp ChAd63 CS.

Administration of MVA CS

The first volunteer to receive 2 x 10^8 pfu MVA CS (group 1B) will vaccinated alone. In the absence of any safety concerns in this volunteer at least 72 hours after vaccination, two further volunteers in group 1B will be vaccinated with 2 x 10^8 pfu MVA CS. In the absence of any safety concerns in these two vaccines at least 72 hours post vaccination, the remaining volunteers in group 1B and group 2B will be vaccinated with 2 x 10^8 pfu MVA CS.

5.3 Minimising environmental contamination with Genetically Modified Organisms (GMO)

The study will be performed in accordance with UK Genetically Modified Organisms (Contained Use) Regulations (2000) and Ireland's GMO (Deliberate Release) Regulations, S.I. No. 500, 2003. GMO authorisation for deliberate release is obtained from the Irish Environmental Protection Agency (EPA) and approval for use in this trial is sought from the Irish Medicines Board (IMB). In order to minimise dissemination of the recombinant vectored vaccine virus into the environment, the inoculation site will be covered with a dressing after immunisation. This should absorb any virus that may leak out through the needle track. The dressing will be removed from the injection site after 30 minutes (+/- 5 minutes) and will be disposed as GMO waste by autoclaving, in accordance with the relevant local SOPs.

5.4 Vaccine Supply

ChAd63 CS will be supplied to the site by the Clinical Biomanufacturing Unit, University of Oxford, where the vaccine is formulated, vialed and labelled for investigational use only.

MVA CS will be supplied to the Clinical Biomanufacturing Unit, University of Oxford, by Impfstoffwerk Dessau-Tornau (IDT) Biologika GmbH, Germany where the vaccine is formulated and vialed. It will labelled for investigational use only by the CBF, who will then transfer the vaccine to site.

All vaccines will be certified for release by a qualified person (QP) at the CBF, University of Oxford. The vaccines will be shipped on dry ice directly from the CBF to the RCSI and a temperature monitoring logger will be enclosed to ensure cold chain verification.

6. RECRUITMENT AND WITHDRAWAL OF TRIAL VOLUNTEERS

6.1 Volunteers

Volunteers may be recruited by use of an advertisement +/- registration form formally approved by the ethics committee and distributed, posted or presented in the following places:

· In public places with the agreement of the owner / proprietor

· In newspapers or other literature for circulation

- On radio via announcements

· On a website operated by our group or with the agreement of the owner or operator (including on-line recruitment through our web-site)

· By e-mail distribution to a group or list only with the express agreement of the network administrator or with equivalent authorisation

· On stalls or stands at exhibitions or fairs or via lectures at public meetings or educational events

6.2 Informed Consent

All volunteers will sign and date the informed consent form before any study specific procedures are performed. The information sheet will be made available to the volunteer at least 24 hours prior to the screening visit. At the screening visit, the volunteer will be fully informed of all aspects of the trial, the potential risks and their obligations. The following general principles will be emphasised:

- Participation in the study is entirely voluntary
- Refusal to participate involves no penalty or loss of medical benefits
- The volunteer may withdraw from the study at any time
- The volunteer is free to ask questions at any time to allow him or her to understand the purpose of the study and the procedures involved
- The study involves research of investigational vaccines. The possibility of increased local and systemic reactions and other potential vaccine associated events will be stressed. Also that long-term effects on the immune system functions are unknown.
- There is no direct benefit from participating
- The volunteer's GP will be contacted to corroborate their medical history and confirm that the volunteer is eligible to take part in the study. UK volunteers will only be enrolled in the study if written or verbal information regarding the volunteer's medical history is obtained from the GP.
- UK volunteers will be registered on the TOPS database (The Overvolunteering Prevention System).
- Separate consent for purposes of genetic testing must be obtained. Not consenting to this aspect of the study will not effect volunteers enrolment in the rest of the study.

The aims of the study and all tests to be carried out will be explained. The volunteer will be given the opportunity to ask about details of the trial, and will then have time to consider whether or not to participate. If they do decide to participate, they will sign and date two copies of the consent form, one for them to take away and keep, and one to be stored in the CRF. In addition, if they so wish, they will sign and date two copies of the DNA testing consent form. These forms will also be signed and dated by the Investigator. No trial specific examinations or tests may be performed until the volunteer has consented to participate in the study, and has signed the trial specific consent form.

6.3 Inclusion and Exclusion Criteria

Inclusion Criteria

The volunteer must satisfy all the following criteria to be eligible for the study:

- Healthy adults aged 18 to 50 years
- Able and willing (in the Investigator's opinion) to comply with all study requirements
- Willing to allow the investigators to discuss the volunteer's medical history with their General Practitioner
- Women only: Must practice continuous effective contraception for the duration of the study.
- Agreement to refrain from blood donation during the course of the study and for 6 months after the end of their involvement in the study.
- Written informed consent.

Exclusion Criteria

The volunteer may not enter the study if any of the following apply:

- History of clinical *P. falciparum* malaria
- Travel to a malaria endemic region during the study period or within the preceding six months with a significant risk of malaria exposure.
- Participation in another research study involving an investigational product in the 30 days preceding enrolment, or planned use during the study period.
- Prior receipt of an investigational malaria vaccine or any other investigational vaccine likely to impact on interpretation of the trial data.
- Administration of immunoglobulins and/or any blood products within the three months preceding the planned administration of the vaccine candidate.
- Any confirmed or suspected immunosuppressive or immunodeficient state, including HIV infection; asplenia; recurrent, severe infections and chronic (more than 14 days) immunosuppressant medication within the past 6 months (inhaled and topical steroids are allowed)
- Pregnancy, breast feeding or intention to become pregnant during the study
- History of allergic disease or reactions likely to be exacerbated by any component of the vaccine e.g. egg products, Kathon.
- History of clinically significant contact dermatitis.
- Any history of anaphylaxis post vaccination or any serious reaction following vaccination.
- History of cancer (except basal cell carcinoma of the skin and cervical carcinoma in situ).
- History of migraine headache.
- History of serious psychiatric condition that may affect participation in the study.
- Any other serious chronic illness requiring hospital specialist supervision. Use of regular medications such as antihypertensives would not necessarily result in exclusion.
- Suspected or known current alcohol abuse as defined by an alcohol intake of greater than 42 units every week or Carbohydrate Deficient Transferrin (CDT) >3%.
- Suspected or known injecting drug abuse in the 5 years preceding enrolment.

- Suspected or known use of opiates, cocaine, amphetamines, benzodiazepines or marijuana.
- Seropositive for hepatitis B surface antigen (HBsAg).
- Seropositive for hepatitis C virus (antibodies to HCV).
- Any clinically significant abnormal finding on biochemistry or haematology blood tests, urinalysis or clinical examination.
- Any other significant disease, disorder or finding which may significantly increase the risk to the volunteer because of participation in the study, affect the ability of the volunteer to participate in the study or impair interpretation of the study data.
  in a particularly dependent relationship with the investigator by way of occupation or otherwise, which in the investigators opinion places the volunteer in a vulnerable population.

Re-vaccination exclusion criteria

The following AEs associated with vaccine immunisation constitute absolute contraindications to further administration of an IMP to a volunteer. If any of these events occur during the study, the subject will be withdrawn from the trial and followed up by the clinical team or their GP until resolution or stabilisation of the event;

- Anaphylactic reaction following administration of vaccine
- Any serious reaction following vaccination
- Pregnancy

The following adverse events constitute contraindications to administration of vaccine at that point in time; if any one of these adverse events occurs at the time scheduled for vaccination, the subject may be vaccinated at a later date, or withdrawn, at the discretion of the investigator;

- Acute disease at the time of vaccination. (Acute disease is defined as the presence of a moderate or severe illness with or without fever.) All vaccines can be administered to persons with a minor illness such as diarrhoea, mild upper respiratory infection with or without low-grade febrile illness, *i.e.*, temperature of <37.5°C (99.5°F).
- Temperature of ≥37.5°C (99.5°F) at the time of vaccination.

6.4 Withdrawal of Volunteers

Volunteers may withdraw or be withdrawn for any of the reasons given below. The reason for withdrawal will be recorded in the CRF. If withdrawal is due to an AE, appropriate follow-up visits or medical care will be arranged with the volunteer's permission, until the AE has resolved or stabilised. Any volunteer who is withdraw or are withdrawn, post vaccination, will be invited to attend for all scheduled safety bloods and review as per protocol. Any volunteer who is withdrawn from the study may be replaced, if that is possible within the specified time frame. The Local Safety Monitor (LSM) may recommend withdrawal of volunteers.

6.5 Discontinuation Criteria

In accordance with the principles of the current revision of the Declaration of Helsinki (updated 2008) and any other applicable regulations, a volunteer has the right to withdraw from the study at any time and for any reason, and is not obliged to give his or her reasons for doing so. The Investigator may withdraw the volunteer at any time in the interests of the volunteer's health and well-being. In addition the volunteer may withdraw/be withdrawn for any of the following reasons:

- Administrative decision by the Investigator

- Ineligibility (either arising during the study or retrospectively, having been overlooked at screening)
- Significant protocol deviation
- Volunteer non-compliance with study requirements
- An AE which requires discontinuation of the vaccination regimen or results in inability to continue to comply with study procedures.

6.6 Pregnancy

Should a volunteer become pregnant during the trial, she will be followed up as other volunteers and in addition will be followed until pregnancy outcome, with the volunteer's permission. We will not routinely perform venepuncture on such volunteers.

7. TREATMENT OF TRIAL VOLUNTEERS

7.1 Study procedures

Procedures will be performed on the visit time points indicated in the schedule of procedures (Tables 5 & 6). Additional procedures or laboratory tests may be performed, at the discretion of the investigators if clinically indicated e.g. urine microscopy in the event of positive urinalysis, repeat of an abnormal blood test.

Observations

Pulse, blood pressure and temperature will be measured and documented at screening, immediately pre-vaccination, at visits scheduled 1 day post vaccination, and at any other time point if felt necessary by the clinical team (Tables 5 & 6).

Blood Tests

Blood will be drawn as scheduled (Tables 5 & 6) for the following laboratory tests:

- **Haematology;** Full Blood Count
- **Biochemistry;** Sodium, Potassium, Urea, Creatinine, Albumin, Bilirubin, Alanine transaminase and alkaline phosphatase
- **Carbohydrate Deficient Transferrin;** screeing test for chronic alcoholism.
- **Diagnostic serology;** HBsAg, HCV antibodies, HIV antibodies (Counselling will be given prior to testing blood for these blood-borne viruses)
- **Immunology;** Human Leukocyte Antigen (HLA) typing and *ex vivo* Elispot assays for interferon gamma. Other exploratory immunological assays including flow cytometry assays, cytokine analysis, functional antibody assays including IFA, anti-adenovirus antibodies, DNA analysis of genetic polymorphisms potentially relevant to vaccine immunogenicity, RNA analysis by either microarray or RNA Seq or other methods and *in vitro* growth inhibition assays amongst others may be performed at the discretion of the investigators.

Urinalysis

- Urine will be tested for the presence of clinically significant proteinuria, glucosuria or haematuria and drug use (Cocaine, Opiates, Benzodiazepines, Amphetamines and Marijuana).

at screening. For female volunteers only, urine will be tested for beta-human chorionic gonadotrophin (βHCG) at screening and immediately prior to each vaccination.

Vaccinations

Before each vaccination, the on-going eligibility of the volunteer will be reviewed. The vaccine will be administered as described in section 5.2. The injection site will be covered with a sterile dressing and the volunteer will stay in the clinical area for 30 minutes (+/- 5 minutes) post vaccination. In the case of the first 3 subjects to receive each dosing (ChAd63 CS and MVA CS respectively) an observation time of 12 hours post vaccination will be applied. The sterile dressing will be removed and injection site inspected in all groups at 30 minutes (+/- 5 minutes) post vaccination. An oral thermometer, tape measure and a 7 day diary card for solicited AEs will be given to each volunteer along with the emergency 24 hour telephone number to contact the on call study physician if needed. Volunteers will be advised that they may experience pain at the injection site and that use of paracetamol or Non Steroidal Anti inflammatories is permitted if they desire. Volunteers will be asked to record all medications used independently by them in the diary card provided.

7.2 Clinical Reviews

The clinical reviews and procedures will be undertaken by one of the clinical team. The procedures to be included in each visit are documented in the schedule specific to each group (Tables 5 & 6). Each review is assigned a time point and a window period, within which the review will be conducted. The first clinical review following any vaccination will take place at 24 hours (Tables 5&6). In respect of the first vaccinee, no further volunteers can be vaccinated until the first review has been satisfactorily completed on the 1st vaccinee for each vaccine. A final review will take place at 6 months post vaccination, this can be in the form of clinic visit or telephone contact.

All potential volunteers will have a screening visit which may take place up to 90 days prior to vaccination. Informed consent will be taken before screening as described above. If consent is obtained, the screening procedures indicated in the schedule of procedures will be undertaken. To avoid unnecessary additional venepuncture, if the appropriate blood test results for screening are available for the same volunteer from a screening visit for another vaccine study, these results may be used for assessing eligibility (provided the results date within the 3 months preceding enrolment in VAC038).

Abnormal clinical findings from the medical history, examination or blood tests at any point in the study will be assessed by the trial clinician. Any abnormal findings deemed untoward medical occurrences will be recorded as AEs. Findings may be reassessed to determine whether the abnormal finding is an isolated occurrence or a persisting abnormality. If an abnormal finding remains clinically significant, the volunteer will be informed and appropriate medical follow-up and care arranged as appropriate, with the permission of the volunteer. Decisions to exclude the volunteer from the enrolling in the trial or to withdraw a volunteer from the trial will be at the discretion of the Investigator, following procedures for adverse events as described in section 9.

Table 5: *Schedule of clinical reviews for Groups 1A & 2A*

	S	ChAd63 CS						
Review No.	1	2	3	4	5	6	7	8†
Timeline (days)		0	1	14	28	56	90	180
Window (days)	-90		**	±2	±7	±7	±14	+14
Inclusion / Exclusion criteria	X	X						
Informed consent	X							
Medical History	X	(x)	(x)	(x)	(x)	(x)	(x)	
Physical Examination	X	(x)	(x)	(x)	(x)	(x)	(x)	
Urinalysis	X							
β-HCG urine test	X	X				X		
Review contraindications	X	X				X		
Vaccination		X						
Vital signs	X	X	X	(x)	(x)	(x)	(x)	
Local & systemic AEs assessed		X	X	X	X	X	X	X
Diary cards provided		X						
Diary cards collected				X				
HLA typing (mL)		4						
HBV,HCV,HIV (mL)	5							
Haematology (mL)	2			2	2	2	2	
Biochemistry* (mL)	4			4	4	4	4	
Carbohydrate Deficient Transferrin	5							
Exploratory immunology		70	13	70	70	70	70	
Blood volume per visit (mL)	16	74	13	76	76	76	76	
Cummulative blood volume (mL)	16	90	103	179	255	331	407	

S = screening visit, **V** = vaccination visit, **(x)** = If necessary (Windows refer to time since last visit) ** Biochemistry will include Sodium, Potassium, Urea, Creatinine & Liver Function Tests. **Window: -12 hours/ +48 hours. † can be via telephone*

Table 6: *Schedule of clinical reviews for Groups 1B & 2B*

	S	ChAd63 CS				MVA CS					
Review No.	1	2	3	4	5	6	7	8	9	10	11†
Timeline (days)		0	1	14	28	56	57	63	84	140	180
Window (days)	-90		**	±2	±7	±7	**	±2	±7	±14	+14
Inclusion / Exclusion criteria	X	X				X					
Informed consent	X										
Medical History	X	(x)	(x)	(x)	(x)	(x)	(x)	(x)	(x)	(x)	
Physical Examination	X	(x)	(x)	(x)	(x)	(x)	(x)	(x)	(x)	(x)	
Urinalysis	X										
β-HCG urine test	X	X				X					
Review contraindications	X	X				X					
Vaccination		X				X					
Vital signs	X	X	X	(x)	(x)	X	X	(x)	(x)	(x)	
Local & systemic AEs assessed		X	X	X	X	X	X	X	X	X	X
Diary cards provided		X				X					
Diary cards collected				X				X			
HLA typing (mL)		4									
HBV,HCV,HIV (mL)	5										
Haematology (mL)	2			2	2	2		2	2	2	
Biochemistry* (mL)	4			4	4	4		4	4	4	
Carbohydrate Deficient Transferrin	5										
Exploratory immunology		70	13	70	70	70	13	70	70	70	
Blood volume per visit (mL)	16	74	13	76	76	76	13	76	76	76	
Cummulative blood volume (mL)	16	90	103	179	255	331	344	420	496	572	

S = screening visit, **V** = vaccination visit, **(x)** = If necessary (Windows refer to time since last visit) ** Biochemistry will include Sodium, Potassium, Urea, Creatinine & Liver Function Tests. **Window: -12 hours/ +48 hours. † can be via telephone.*

Table 7: Total Blood Drawn During Study:

Group	Maximum Total Blood Donated During Study	Max Duration of Involvement in Study (approx)
1A & 2A	407 mls	6 months
1B & 2B	572 mls	6 months

8. ASSESSMENT OF SCIENTIFIC OBJECTIVES

8.1 PRIMARY EVALUATION CRITERIA

- Safety of the vaccine regimens will be assessed by analysing actively and passively collected data on AEs from diary cards, clinical review of volunteers and laboratory measurements.

8.2 SECONDARY EVALUATION CRITERIA

- Immunological assays will be conducted according to the procedures established in the test laboratories. With the volunteers' written informed consent, any leftover cells and serum from UK volunteers will be frozen for up to 15 years for future immunological analysis of malaria-specific responses (A Study of Exploratory Immunological Assays to Provide a Laboratory Based Correlate of Protection From Malaria; OXREC Number: 06/Q1606/123)
- The following parameters will be considered evidence of the impact of vaccination in inducing malaria-specific immune responses. Other laboratory investigations including microarray analysis may be performed.

(A) Interferon gamma CS peptide ELISPOT.
(B) Flow cytometry to measure T cell responses to CS
(C) Antibody response. ELISA will be used to assess the levels of anti-CS antibodies.

9. ASSESSMENT OF SAFETY

Safety will be assessed by the frequency, incidence and nature of adverse events and serious adverse events arising during the study.

Definitions

Adverse Event (AE)

An AE is any untoward medical occurrence in a volunteer, including a dosing error, which may occur during or after study vaccination and does not necessarily have to have a causal relationship with vaccination. An AE can therefore be any unfavourable and unintended sign (including an abnormal laboratory finding), symptom or disease temporally associated with study vaccination, whether or not considered related to study vaccination.

Adverse Drug Reaction (ADR)

An ADR is any untoward or unintended response to a medicinal product. This means that a causal relationship between the study medication and an AE is at least a reasonable possibility, i.e., the relationship cannot be ruled out.

Unexpected Adverse Reaction

An unexpected adverse reaction is where the nature or severity is not consistent with the Investigator's Brochure.

Serious Adverse Event (SAE)

An SAE is an AE that results in any of the following outcomes, whether or not considered related to the vaccine.

- Death (i.e., results in death from any cause at any time)
- Life-threatening event (i.e., the volunteer was, in the view of the investigator, at immediate risk of death from the event that occurred). This does not include an AE that, if it occurred in a more serious form, might have caused death.
- Persistent or significant disability or incapacity (i.e. substantial disruption of one's ability to carry out normal life functions).
- Hospitalisation, regardless of length of stay, even if it is a precautionary measure for continued observation. Hospitalisation (including inpatient or outpatient hospitalization for an elective procedure) for a pre-existing condition that has not worsened unexpectedly does not constitute a serious AE.
- An important medical event (that may not cause death, be life threatening, or require hospitalization) that may, based upon appropriate medical judgment, jeopardize the volunteer and/or require medical or surgical intervention to prevent one of the outcomes listed above. Examples of such medical events include allergic reaction requiring intensive treatment in an emergency room or clinic, blood dyscrasias, or convulsions that do not result in inpatient hospitalization.
- Congenital anomaly or birth defect.

Suspected Unexpected Serious Adverse Reactions (SUSARs)

A SUSAR is a SAE that is unexpected and thought to be possibly, probably or definitely related to the investigational product. Reports of any SUSAR will be sent to the REC,

regulatory authority and sponsor according to the sponsor's SOP and national regulatory requirements. Administration of further vaccines within the trial will be suspended until a safety review is convened.

Foreseeable Adverse Drug Reactions

Expected local reactions to the vaccine will be recorded as AEs. These include injection site pain, erythema, warmth, swelling or itching. Expected systemic reactions to the vaccine will be recorded as in the CRF as AEs. These include myalgia, arthralgia, fatigue, malaise, nausea, fever, feverishness and headache.

Foreseeable Serious Adverse Events

No IMP related serious adverse events are expected in this study. If an SAE occurs it will be reported as described in section 9.3 below.

9.1 Causality Assessment

For every AE an assessment of the relationship of the event to the administration of the vaccine will be undertaken. An intervention-related AE refers to an AE for which there is a possible, probable or definite relationship to administration of a vaccine. An interpretation of the causal relationship of the intervention to the AE in question will be made, based on the type of event; the relationship of the event to the time of vaccine administration; and the known biology of the vaccine action (table 8).

Table 8: *Guidelines for assessing the relationship of vaccine administration to an AE*

0	**No Relationship**	No temporal relationship to study product ***and***<br>Alternate aetiology (clinical state, environmental or other interventions); ***and***<br>Does not follow known pattern of response to study product
1	**Unlikely**	Unlikely temporal relationship to study product ***and***<br>Alternate aetiology likely (clinical state, environmental or other interventions) ***and***<br>Does not follow known typical or plausible pattern of response to study product.
2	**Possible**	Reasonable temporal relationship to study product; ***or***<br>Event not readily produced by clinical state, environmental or other interventions; ***or***<br>Similar pattern of response to that seen with other vaccines
3	**Probable**	Reasonable temporal relationship to study product; ***and***<br>Event not readily produced by clinical state, environment, or other interventions ***or***<br>Known pattern of response seen with other vaccines
4	**Definite**	Reasonable temporal relationship to study product; ***and***<br>Event not readily produced by clinical state, environment, or other interventions; ***and***<br>Known pattern of response seen with other vaccines

9.2 Reporting Procedures for Adverse Events

All AEs occurring during the study observed by the investigator or reported by the patient will be recorded in the CRF. AEs that result in a patient's withdrawal from the study or that are present at the end of the study will be followed up (with the volunteer's permission) until a satisfactory resolution or stabilisation occurs, or until a non-study related causality is assigned.

The severity of clinical and laboratory adverse events will be assessed according to the scales in Tables 9, 10 & 11.

Laboratory values which fall outside the reference range of the local laboratory processing samples will be deemed laboratory abnormalities. These abnormalities will be assessed by the trial clinician. If deemed an untoward medical occurrence, this abnormality will be documented as an AE and ascribed a severity grading (Table 10). Laboratory abnormalities that are not considered AEs will be collated by the investigators and included in the end of study report.

Table 9: *Severity grading criteria for adverse events*

Adverse Event	Grade	Intensity
Pain at injection site	1	Pain that is easily tolerated
	2	Pain that interferes with daily activity
	3	Pain that prevents daily activity
Erythema at injection site*	1	>3 - ≤50 mm
	2	>50 - ≤100 mm
	3	>100 mm
Swelling at injection site	1	>3 - ≤50 mm
	2	>50 - ≤100 mm
	3	>100 mm
Fever (oral)	1	37.6°C - 38.0°C
	2	>38.0°C – 39.0°C
	3	>39.0°C
Headache	1	Headache that is easily tolerated
	2	Headache that interferes with daily activity
	3	Headache that prevents daily activity
Nausea	1	Nausea that is easily tolerated
	2	Nausea that interferes with daily activity
	3	Nausea that prevents daily activity
Malaise	1	Malaise that is easily tolerated
	2	Malaise that interferes with daily activity
	3	Malaise that prevents daily activity
Myalgia	1	Myalgia that is easily tolerated
	2	Myalgia that interferes with daily activity
	3	Myalgia that prevents daily activity

Arthralgia	1	Joint pain that is easily tolerated
	2	Joint pain that interferes with daily activity
	3	Joint pain that prevents daily activity
Urticaria	1	Requiring no medications
	2	Requiring oral or topical treatment or IV medication or steroids for <24 hours
	3	Requiring IV medication or steroids for >24 hours

**erythema ≤3mm is an expected consequence of skin puncture and will therefore not be considered an adverse event*

Table 10: *Severity grading criteria for laboratory abnormalities*

**Laboratory Test**	**Grade 1**	**Grade 2**	**Grade 3**
Hgb (female) – decrease from testing laboratory LLN in gm/dl	>1.0 - <1.5	≥1.5 & <2.0	≥2.0
Hgb (male) – decrease from testing laboratory LLN in gm/dl	≥1.5 & <2.0	≥2.0 & <2.5	≥2.5
Absolute neutrophil count (ANC, cells/mm^3)	1000-1499	500-999	<500
Leukopenia (WBC, cells/mm^3)	<3500 - ≥2500	<2500 - ≥1500	<1500
Platelets (cells/mm^3)	125,000 – 135,000	100,000 – 124,000	20,000-99,000
Bilirubin – when accompanied by any increase in Liver Function Test increase by factor	1.1 – 1.25 x ULN	1.26 – 1.5 x ULN	1.51 – 1.75 x ULN
ALT	1.25 – 2.5 x ULN	>2.6 – 5.0 x ULN	>5.0 x ULN
Creatinine	1.1 – 1.5 x ULN	>1.6 – 3.0 x ULN	>3.0 x ULN
Urine protein	2+ or 0.5-1 gm loss/day	3+ or 1-2 gm loss/day	4+ or >2 gm loss/day
Hematuria	2+ confirmed by 5-10 rbc/hpf	3+ confirmed by >10 rbc/hpf	gross, with or without clots, OR red blood cell casts

Table 11: *Functional scale for assessing the severity of AEs*

Scale	Description	Definition
**1**	Mild	Awareness of a symptom but the symptom is easily tolerated
**2**	Moderate	Discomfort enough to cause interference with usual activity
**3**	Severe	Incapacitating; unable to perform usual activities; requires absenteeism or bed rest

9.3 Reporting Procedures for Serious Adverse Events

The event will be documented accurately and national & sponsor notification deadlines and reporting procedures adhered to (see SOP). In addition to the expedited reporting above, the investigator shall submit once a year throughout the study or, on request, a safety report to the sponsor (CTRG), the regulatory authority and REC.

9.4 Reporting Procedures for SUSARs

The Chief Investigator will report all SUSARs to the sponsor (CTRG), The Medicines and Healthcare products Regulatory Agency, The Irish Medicines Board (IMB) and the RECs concerned within required timelines. Fatal or life-threatening SUSARs must be reported within 7 days and all other SUSARs within 15 days. The Chief Investigator will also inform all investigators concerned of relevant information about SUSARs.

For all deaths, available autopsy reports and relevant medical reports will be made available for reporting to the relevant authorities.

9.5 Procedures to be followed in the event of abnormal findings

Any abnormal findings deemed untoward medical occurrences will be recorded as AEs. Findings may be reassessed to determine whether the abnormal finding is an isolated occurrence or a persisting abnormality. If an abnormal finding remains clinically significant, the volunteer will be informed and appropriate medical follow-up and care arranged as appropriate, with the permission of the volunteer. Decisions to exclude the volunteer from the enrolling in the trial or to withdraw a volunteer from the trial will be at the discretion of the Investigator, following procedures for adverse events as described in section 9.

9.6 DSMB

A Drug safety monitoring board (DSMB) will provide real-time safety oversight. The DSMB will review SAEs deemed possibly, probably or definitely related to vaccination. The DSMB will be notified within 1 working day of the investigators being aware of their occurrence. The DSMB has the power to terminate the study if deemed necessary following a vaccine-related SAE. The DSMB will review the data before there is a dose escalation of ChAd63 CS from 5×10^9 to 5×10^{10}. The DSMB will be contacted for advice and independent review in the following situations:

- Following any SAE deemed to be possibly, probably, or definitely related to the study vaccine.
- Any other situation where the Investigator feels independent advice or review is important.

9.7 Safety Profile Review

The safety profile will be assessed on an on-going basis by the investigators. An internal safety group will also review safety issues and SAEs as they arise.

10. STATISTICS

This is an observational, un-blinded, non-randomised safety study. The number of vaccinated subjects in each group recruited for the study will be 4-8. This sample size should allow determination of the magnitude of the outcome measures, especially of serious and severe adverse events, rather than aiming to obtain statistical significance. Data analysis will consist primarily of descriptive summaries for treatment groups. For primary and secondary endpoints descriptive summaries and plots over the time course for both individual patient results and groups will be presented. Due the small number of volunteers in this study, all volunteers receiving the same dose of a given vaccine will be pooled for analysis. Where appropriate highly skewed data will be log-transformed and presented as geometric means with 95% confidence intervals.

11. QUALITY CONTROL AND QUALITY ASSURANCE PROCEDURES

Investigator procedures
Approved site-specific SOPs will be used at all clinical and laboratory sites.

Monitoring
Monitoring will be performed according to ICH Good Clinical Practice (GCP) by the external monitor Appledown Clinical Research Ltd. Following written standard operating procedures, the monitors will verify that the clinical trial is conducted and data are generated, documented and reported in compliance with the protocol, GCP and the applicable regulatory requirements. The investigator sites will provide direct access to all trial related source data/documents and reports for the purpose of monitoring and auditing by the sponsor and inspection by local and regulatory authorities.

Modification to protocol
No amendments to this protocol will be made without consultation with, and agreement of, the Sponsor. Any amendments to the trial that appear necessary during the course of the trial must be discussed by the Investigator and Sponsor concurrently. If agreement is reached concerning the need for an amendment, it will be produced in writing by the Chief Investigator and will be made a formal part of the protocol following ethical and regulatory approval.

An administrative change to the protocol is one that modifies administrative and logistical aspects of a protocol but does not affect the subjects' safety, the objectives of the trial and its progress. An administrative change does not require REC or regulatory approval.

The Investigator is responsible for ensuring that changes to an approved trial, during the period for which regulatory and REC approval has already been given, are not initiated without regulatory and REC review and approval except to eliminate apparent immediate hazards to the subject.

Protocol deviation

Any deviations from the protocol will be documented in a protocol deviation form and filed in the site trial master file.

Audit & inspection

The QA manager will conduct internal audits to check that the trial is being conducted, data recorded, analyzed and accurately reported according to the protocol, sponsor's SOPs and in compliance with ICH GCP. The audits will also include laboratory activities according to an agreed audit schedule. The internal audits will supplement the external monitoring process and will review processes not covered by the external monitor.

The sponsor and trial sites may carry out audit to ensure compliance with the protocol, GCP and appropriate regulations. GCP inspections may also be undertaken by the regulatory authority to ensure compliance with protocol and national regulations. The sponsor will assist in any inspections.

Serious Breaches

The UK Medicines for Human Use (Clinical Trials) Regulations contain a requirement for the notification of "serious breaches" to the regulatory authority within 7 days of the sponsor becoming aware of the breach.

A serious breach is defined as "A breach of GCP or the trial protocol which is likely to effect to a significant degree –

(a) the safety or physical or mental integrity of the subjects of the trial; or
(b) the scientific value of the trial".

In the event that a serious breach is suspected the sponsor will be informed as soon as possible and in turn will notify the MHRA and the IMB within 7 days A copy of this notification will also be forwarded to the Ethics committees.

Trial Progress

The progress of the trial will be overseen by the Chief Investigator.

12. ETHICS

12.1 Declaration of Helsinki

The Investigator will ensure that this study is conducted according to the principles of the current revision of the Declaration of Helsinki 2008.

12.2 ICH Guidelines for Good Clinical Practice

The Investigator will ensure that this study is conducted in full conformity to Medicine for Human use (clinical trials) Regulations 2004 and its amendments and with the ICH guidelines for GCP (CPMP/ICH/135/95) July 1996. The trial will also comply with the European Communities (Clinical Trials on Medicinal Products for Human Use) Regulations, 2004 [S.I. 190 of 2004].

12.3 Informed Consent

Written, informed consent will be obtained, as described above.

12.4 Research Ethics Committee (REC)

A copy of the protocol, proposed informed consent form, other written volunteer information and the proposed advertising material will be submitted to the local RECs for written approval. The Investigator will submit and, where necessary, obtain approval from the local RECs for all subsequent substantial amendments to the protocol and informed consent document. The Investigator will notify deviations from the protocol or SAEs occurring at the site to the sponsor and will notify the local RECs of these if necessary in accordance with local procedures.

12.5 Volunteer Confidentiality

All data will be anonymised; volunteer data will be identified by a unique study number in CRF and database. Separate confidential files containing identifiable information will be stored in secured locations. Only the sponsor representative, investigators, the clinical monitor, the local RECs and the regulatory authorities will have access to the records. Photographs taken of vaccination sites (if required, with the volunteer's written, informed consent) will not include the volunteer's face and will be identified by the volunteer's trial specific identification number only. Once developed, photographs will be stored as confidential records, as above. This material may be shown to other professional staff, used for educational purposes, or included in a scientific publication.

13. DATA HANDLING AND RECORD KEEPING

13.1 Data Handling

The Chief Investigator will be the data manager with responsibility for delegating the receiving, entering, cleaning, querying, analysing and storing all data that accrues from the study. The investigators will enter the data into the volunteers' CRFs, which will be in a paper and/or electronic format. This includes safety data, laboratory data and outcome data.

13.2 Record Keeping

The investigators will maintain appropriate medical and research records for this trial in compliance with ICH E6 GCP and regulatory and institutional requirements for the protection of confidentiality of volunteers. The Chief Investigator, co-investigators and clinical research nurses will have access to records. The investigators will permit authorized representatives of the sponsor(s), regulatory agencies and the monitors to examine (and when required by applicable law, to copy) clinical records for the purposes of quality assurance reviews, audits and evaluation of the study safety and progress.

13.3 Source Data and Case Report Forms (CRFs)

All protocol-required information will be collected in CRFs designed by the investigator. All source documents will be filed in the CRF. Source documents are original documents, data, and records from which the volunteer's CRF data are obtained. For this study these will include, but are not limited to; volunteer consent form, blood results, GP response letters, laboratory records, diaries, and correspondence. In the majority of cases, CRF entries will be considered source data as the CRF is the site of the original recording (i.e. there is no other written or electronic record of data). In this study this will include, but is not limited to medical history, medication records, vital signs, physical examination records, urine assessments, blood results, adverse event data and details of vaccinations. All source data and volunteer CRFs will be stored securely.

13.4 Data Protection

The study protocol, documentation, data and all other information generated will be held in strict confidence. No information concerning the study or the data will be released to any unauthorized third party, without prior written approval of the sponsor.

14. FINANCING AND INSURANCE

14.1 Financing

The study will be funded primarily by a grant from the European Vaccine Initiative (EVI) with further support from other research grants from the Wellcome Trust, the National Institute of Health Research and the Medical Research Council, held by Professor Adrian Hill.

14.2 Insurance

Negligent Harm: Indemnity and/or compensation for negligent harm arising specifically from an accidental injury for which the University is legally liable as the Research Sponsor will be covered by the University of Oxford.

Non-Negligent Harm: Indemnity and/or compensation for harm arising specifically from an accidental injury, and occurring as a consequence of the Research Subjects' participation in the trial for which the University is the Research Sponsor will be covered by the University of Oxford.

14.3 Compensation

Volunteers will be compensated for their time and for the inconvenience caused by procedures as below.

UK volunteers

- Travel expenses	£6* per visit
- Inconvenience of blood tests:	£6 per blood donation
- Time required for visit:	£15 per hour

Republic of Ireland volunteers:

- Travel expenses	€10* per visit
- Inconvenience of blood tests:	€10 per blood donation
- Time required for visit:	€10 per hour

*Guide value – this may change depending on individual volunteer's travel arrangements.

15. REFERENCES

1. Okiro EA, Hay SI, Gikandi PW, et al. The decline in paediatric malaria admissions on the coast of Kenya. Malar J 2007;6:151.

2. WHO. World Malaria Report. World Health Organisation 2009.

3. Sachs J, Malaney P. The economic and social burden of malaria. Nature 2002;415:680-5.

4. White NJ. Antimalarial drug resistance. J Clin Invest 2004;113:1084-92.

5. Das P, Horton R. Malaria elimination: worthy, challenging, and just possible. Lancet 2010;376:1515-7.

6. Cohen J, Nussenzweig V, Nussenzweig R, Vekemans J, Leach A. From the circumsporozoite protein to the RTS, S/AS candidate vaccine. Hum Vaccin 2010;6:90-6.

7. Hoffman SL, Goh LM, Luke TC, et al. Protection of humans against malaria by immunization with radiation-attenuated Plasmodium falciparum sporozoites. J Infect Dis 2002;185:1155-64.

8. Greenwood B. Immunological correlates of protection for the RTS,S candidate malaria vaccine. Lancet Infect Dis 2011;11:75-6.

9. Olotu A, Lusingu J, Leach A, et al. Efficacy of RTS,S/AS01E malaria vaccine and exploratory analysis on anti-circumsporozoite antibody titres and protection in children aged 5-17 months in Kenya and Tanzania: a randomised controlled trial. Lancet Infect Dis 2011;11:102-9.

10. Stoute JA, Slaoui M, Heppner DG, et al. A preliminary evaluation of a recombinant circumsporozoite protein vaccine against Plasmodium falciparum malaria. RTS,S Malaria Vaccine Evaluation Group. N Engl J Med 1997;336:86-91.

11. Stoute JA, Kester KE, Krzych U, et al. Long-term efficacy and immune responses following immunization with the RTS,S malaria vaccine. J Infect Dis 1998;178:1139-44.

12. Aponte JJ, Aide P, Renom M, et al. Safety of the RTS,S/AS02D candidate malaria vaccine in infants living in a highly endemic area of Mozambique: a double blind randomised controlled phase I/IIb trial. Lancet 2007;370:1543-51.

13. Lalvani A, Moris P, Voss G, et al. Potent induction of focused Th1-type cellular and humoral immune responses by RTS,S/SBAS2, a recombinant Plasmodium falciparum malaria vaccine. J Infect Dis 1999;180:1656-64.

14. Schwenk RJ, Richie TL. Protective immunity to pre-erythrocytic stage malaria. Trends Parasitol 2011.

15. Khusmith S, Sedegah M, Hoffman SL. Complete protection against Plasmodium yoelii by adoptive transfer of a CD8+ cytotoxic T-cell clone recognizing sporozoite surface protein 2. Infect Immun 1994;62:2979-83.

16. Romero P, Maryanski JL, Corradin G, Nussenzweig RS, Nussenzweig V, Zavala F. Cloned cytotoxic T cells recognize an epitope in the circumsporozoite protein and protect against malaria. Nature 1989;341:323-6.

17. Schofield L, Villaquiran J, Ferreira A, Schellekens H, Nussenzweig R, Nussenzweig V. Gamma interferon, CD8+ T cells and antibodies required for immunity to malaria sporozoites. Nature 1987;330:664-6.

18. Hoffman SL, Isenbarger D, Long GW, et al. Sporozoite vaccine induces genetically restricted T cell elimination of malaria from hepatocytes. Science 1989;244:1078-81.

19. Weiss WR, Mellouk S, Houghten RA, et al. Cytotoxic T cells recognize a peptide from the circumsporozoite protein on malaria-infected hepatocytes. J Exp Med 1990;171:763-73.

20. Webster D, Dunachie S, McConkey S, et al. Safety of recombinant fowlpox strain 9 and modified vaccinia virus Ankara vaccines against liver-stage *P. falciparum* malaria non-immune volunteers. In press (Vaccine) 2005.

21. McConkey SJ, Reece WH, Moorthy VS, et al. Enhanced T-cell immunogenicity of plasmid DNA vaccines boosted by recombinant modified vaccinia virus Ankara in humans. Nat Med 2003;9:729-35.

22. Dunachie SJ, Walther M, Epstein JE, et al. A DNA prime-modified vaccinia virus ankara boost vaccine encoding thrombospondin-related adhesion protein but not circumsporozoite protein partially protects healthy malaria-naive adults against Plasmodium falciparum sporozoite challenge. Infect Immun 2006;74:5933-42.

23. Webster DP, Dunachie S, Vuola JM, et al. Enhanced T cell-mediated protection against malaria in human challenges by using the recombinant poxviruses FP9 and modified vaccinia virus Ankara. Proc Natl Acad Sci U S A 2005;102:4836-41.

24. Dunachie SJ, Walther M, Vuola JM, et al. A clinical trial of prime-boost immunisation with the candidate malaria vaccines RTS,S/AS02A and MVA-CS. Vaccine 2006;24:2850-9.

25. Walther M, Thompson FM, Dunachie S, et al. Safety, immunogenicity, and efficacy of prime-boost immunization with recombinant poxvirus FP9 and modified vaccinia virus Ankara encoding the full-length Plasmodium falciparum circumsporozoite protein. Infect Immun 2006;74:2706-16.

26. Imoukhuede EB, Berthoud T, Milligan P, et al. Safety and immunogenicity of the malaria candidate vaccines FP9 CS and MVA CS in adult Gambian men. Vaccine 2006;24:6526-33.

27. Bejon P, Peshu N, Gilbert SC, et al. Safety profile of the viral vectors of attenuated fowlpox strain FP9 and modified vaccinia virus Ankara recombinant for either of 2 preerythrocytic malaria antigens, ME-TRAP or the circumsporozoite protein, in children and adults in Kenya. Clin Infect Dis 2006;42:1102-10.

28. Bruna-Romero O, Rocha CD, Tsuji M, Gazzinelli RT. Enhanced protective immunity against malaria by vaccination with a recombinant adenovirus encoding the circumsporozoite protein of Plasmodium lacking the GPI-anchoring motif. Vaccine 2004;22:3575-84.

29. Coppi A, Pinzon-Ortiz C, Hutter C, Sinnis P. The Plasmodium circumsporozoite protein is proteolytically processed during cell invasion. J Exp Med 2005;201:27-33.

30. Singh AP, Buscaglia CA, Wang Q, et al. Plasmodium circumsporozoite protein promotes the development of the liver stages of the parasite. Cell 2007;131:492-504.

31. Bett AJ, Haddara W, Prevec L, Graham FL. An efficient and flexible system for construction of adenovirus vectors with insertions or deletions in early regions 1 and 3. Proc Natl Acad Sci U S A 1994;91:8802-6.

32. Top FH, Jr., Grossman RA, Bartelloni PJ, et al. Immunization with live types 7 and 4 adenovirus vaccines. I. Safety, infectivity, antigenicity, and potency of adenovirus type 7 vaccine in humans. J Infect Dis 1971;124:148-54.

33. Tatsis N, Ertl HC. Adenoviruses as vaccine vectors. Mol Ther 2004;10:616-29.

34. Kobinger GP, Feldmann H, Zhi Y, et al. Chimpanzee adenovirus vaccine protects against Zaire Ebola virus. Virology 2006;346:394-401.

35. Casimiro DR, Chen L, Fu TM, et al. Comparative immunogenicity in rhesus monkeys of DNA plasmid, recombinant vaccinia virus, and replication-defective adenovirus vectors expressing a human immunodeficiency virus type 1 gag gene. J Virol 2003;77:6305-13.

36. Xiang Z, Gao G, Reyes-Sandoval A, et al. Novel, chimpanzee serotype 68-based adenoviral vaccine carrier for induction of antibodies to a transgene product. J Virol 2002;76:2667-75.

37. Catanzaro AT, Koup RA, Roederer M, et al. Phase 1 safety and immunogenicity evaluation of a multiclade HIV-1 candidate vaccine delivered by a replication-defective recombinant adenovirus vector. J Infect Dis 2006;194:1638-49.

38. Harro CD, Robertson MN, Lally MA, et al. Safety and immunogenicity of adenovirus-vectored near-consensus HIV type 1 clade B gag vaccines in healthy adults. AIDS Res Hum Retroviruses 2009;25:103-14.

39. Casimiro DR, Bett AJ, Fu TM, et al. Heterologous human immunodeficiency virus type 1 priming-boosting immunization strategies involving replication-defective adenovirus and poxvirus vaccine vectors. J Virol 2004;78:11434-8.

40. Bruna-Romero O, Gonzalez-Aseguinolaza G, Hafalla JC, Tsuji M, Nussenzweig RS. Complete, long-lasting protection against malaria of mice primed and boosted with two distinct viral vectors expressing the same plasmodial antigen. Proc Natl Acad Sci U S A 2001;98:11491-6.

41. Grabenstein JD, Pittman PR, Greenwood JT, Engler RJ. Immunization to protect the US Armed Forces: heritage, current practice, and prospects. Epidemiol Rev 2006;28:3-26.

42. Farina SF, Gao GP, Xiang ZQ, et al. Replication-defective vector based on a chimpanzee adenovirus. J Virol 2001;75:11603-13.

43. Tatsis N, Tesema L, Robinson ER, et al. Chimpanzee-origin adenovirus vectors as vaccine carriers. Gene Ther 2006;13:421-9.

44. Tatsis N, Blejer A, Lasaro MO, et al. A CD46-binding Chimpanzee Adenovirus Vector as a Vaccine Carrier. Mol Ther 2007.

45. Dudareva M, Andrews L, Gilbert SC, et al. Prevalence of serum neutralizing antibodies against chimpanzee adenovirus 63 and human adenovirus 5 in Kenyan children, in the context of vaccine vector efficacy. Vaccine 2009;27:3501-4.

46. Pinto AR, Fitzgerald JC, Giles-Davis W, Gao GP, Wilson JM, Ertl HC. Induction of CD8+ T cells to an HIV-1 antigen through a prime boost regimen with heterologous E1-deleted adenoviral vaccine carriers. J Immunol 2003;171:6774-9.

47. Mayr A, Stickl H, Muller HK, Danner K, Singer H. [The smallpox vaccination strain MVA: marker, genetic structure, experience gained with the parenteral vaccination and behavior in organisms with a debilitated defence mechanism (author's transl)]. Zentralbl Bakteriol B 1978;167:375-90.

48. Mahnel H, Mayr A. [Experiences with immunization against orthopox viruses of humans and animals using vaccine strain MVA]. Berl Munch Tierarztl Wochenschr 1994;107:253-6.

49. Draper SJ, Heeney JL. Viruses as vaccine vectors for infectious diseases and cancer. Nat Rev Microbiol 2010;8:62-73.

50. Stickl H, Hochstein-Mintzel V, Mayr A, Huber HC, Schafer H, Holzner A. [MVA vaccination against smallpox: clinical tests with an attenuated live vaccinia virus strain (MVA) (author's transl)]. Dtsch Med Wochenschr 1974;99:2386-92.

51. Hanke T, McMichael AJ, Dennis MJ, et al. Biodistribution and persistence of an MVA-vectored candidate HIV vaccine in SIV-infected rhesus macaques and SCID mice. Vaccine 2005;23:1507-14.

52. Akira S, Takeda K, Kaisho T. Toll-like receptors: critical proteins linking innate and acquired immunity. Nat Immunol 2001;2:675-80.

53. Goepfert PA, Elizaga ML, Sato A, et al. Phase 1 safety and immunogenicity testing of DNA and recombinant modified vaccinia Ankara vaccines expressing HIV-1 virus-like particles. J Infect Dis 2011;203:610-9.

54. Howles S, Guimaraes-Walker A, Yang H, et al. Vaccination with a modified vaccinia virus Ankara (MVA)-vectored HIV-1 immunogen induces modest vector-specific T cell responses in human subjects. Vaccine 2010;28:7306-12.

55. Scriba TJ, Tameris M, Mansoor N, et al. Modified vaccinia Ankara-expressing Ag85A, a novel tuberculosis vaccine, is safe in adolescents and children, and induces polyfunctional CD4+ T cells. Eur J Immunol 2010;40:279-90.

56. Berthoud TK, Hamill M, Lillie PJ, et al. Potent CD8+ T-cell immunogenicity in humans of a novel heterosubtypic influenza A vaccine, MVA-NP+M1. Clin Infect Dis 2011;52:1-7.

57. Dangoor A, Lorigan P, Keilholz U, et al. Clinical and immunological responses in metastatic melanoma patients vaccinated with a high-dose poly-epitope vaccine. Cancer Immunol Immunother 2010;59:863-73.
